# Deficits in mitochondrial TCA cycle and OXPHOS precede rod photoreceptor degeneration during chronic HIF activation

**DOI:** 10.1186/s13024-023-00602-x

**Published:** 2023-03-07

**Authors:** Vyara Todorova, Mia Fee Stauffacher, Luca Ravotto, Sarah Nötzli, Duygu Karademir, Lynn J. A. Ebner, Cornelia Imsand, Luca Merolla, Stefanie M. Hauck, Marijana Samardzija, Aiman S. Saab, L. Felipe Barros, Bruno Weber, Christian Grimm

**Affiliations:** 1 Laboratory for Retinal Cell Biology, Department of Ophthalmology, University Hospital Zurich, University of Zurich, Wagistrasse 14, 8952 Schlieren, Switzerland; 2 Institute of Pharmacology and Toxicology and Neuroscience Center Zurich, University and ETH Zurich, Winterthurerstr. 190, 8057 Zurich, Switzerland; 3 Metabolomics and Proteomics Core, Helmholtz Zentrum München, German Research Center for Environmental Health (GmbH), Ingolstädter Landstraße 1, 85764 Munich, Germany; 4 Centro de Estudios Científicos (CECs), Valdivia, Chile; 5 Universidad San Sebastián, Valdivia, Chile

**Keywords:** Hypoxia, Rod photoreceptor, Energy metabolism, Retinal degeneration, Aging, Retinal proteomics, Two-photon microscopy

## Abstract

**Background:**

Major retinal degenerative diseases, including age-related macular degeneration, diabetic retinopathy and retinal detachment, are associated with a local decrease in oxygen availability causing the formation of hypoxic areas affecting the photoreceptor (PR) cells. Here, we addressed the underlying pathological mechanisms of PR degeneration by focusing on energy metabolism during chronic activation of hypoxia-inducible factors (HIFs) in rod PR.

**Methods:**

We used two-photon laser scanning microscopy (TPLSM) of genetically encoded biosensors delivered by adeno-associated viruses (AAV) to determine lactate and glucose dynamics in PR and inner retinal cells. Retinal layer-specific proteomics, *in situ* enzymatic assays and immunofluorescence studies were used to analyse mitochondrial metabolism in rod PRs during chronic HIF activation.

**Results:**

PRs exhibited remarkably higher glycolytic flux through the hexokinases than neurons of the inner retina. Chronic HIF activation in rods did not cause overt change in glucose dynamics but an increase in lactate production nonetheless. Furthermore, dysregulation of the oxidative phosphorylation pathway (OXPHOS) and tricarboxylic acid (TCA) cycle in rods with an activated hypoxic response decelerated cellular anabolism causing shortening of rod photoreceptor outer segments (OS) before onset of cell degeneration. Interestingly, rods with deficient OXPHOS but an intact TCA cycle did not exhibit these early signs of anabolic dysregulation and showed a slower course of degeneration.

**Conclusion:**

Together, these data indicate an exceeding high glycolytic flux in rods and highlight the importance of mitochondrial metabolism and especially of the TCA cycle for PR survival in conditions of increased HIF activity.

**Supplementary Information:**

The online version contains supplementary material available at 10.1186/s13024-023-00602-x.

## Background

The retina is an energetically highly active tissue and relies on a well-functioning blood supply for the delivery of sufficient oxygen and nutrients to meet its metabolic needs [1]. The photoreceptor (PR) cells, residing in the outer retina, are especially energy-demanding and have high oxygen and glucose consumption levels [2–5]. Oxygen reaching the outer retina is mainly used in the photoreceptor inner segments (IS), a specialized cell compartment tightly packed with mitochondria [6]. Here, ATP is produced providing the required energy to maintain ion gradients across the cell membrane by Na^+^/K^+^-ATPase and to sustain vision [7, 8]. Despite the high energy demand, isolated retina produces lactate in the presence of oxygen [9–12]. This incomplete oxidation of glucose under aerobic conditions, or aerobic glycolysis, and the associated high glucose consumption are metabolic adaptations characteristic of anabolic and proliferating cells facilitating the incorporation of nutrients into biomass [13]. Thus, retinal lactate production has been attributed to the highly anabolic PRs possibly supporting the diurnal renewal of the photoreceptor outer segments (OS) [12, 14–16]. A growing body of evidence suggests also metabolic coupling of PRs and the underlying retinal pigmented epithelium (RPE), in which the RPE uses lactate (produced by the PRs) as fuel, sparing glucose for the sake of PRs [16–18].

In the aging human eye, the RPE and the neighbouring choroid undergo a series of morphological changes, including accumulation of intracellular and extracellular deposits, thickening of the Bruch’s membrane, and reduction of choroidal blood flow [19–22]. These morphological alterations may hinder oxygen delivery to the RPE and the outer retina generating a tissue environment where oxygen demand may exceed oxygen availability (hypoxia). Thus, hypoxia is a factor in the development of retinal dysfunction and degeneration during aging [23, 24], as well as in other pathologies including diabetic retinopathy and retinal detachment [25, 26].

An evolutionarily conserved pathway mediated by the hypoxia-inducible transcription factors (HIFs) is crucial for the cellular adaptation to hypoxia [27]. HIFs have been associated with the development of age-related macular degeneration (AMD), providing potential therapeutic targets [28]. Central to the HIF pathway is the von Hippel-Lindau tumor suppressor protein (VHL), that forms an E3 ubiquitin ligase complex together with elongins B and C, cullin 2, and RING box protein 1 [29] and ubiquitinates hydroxylated HIF-alpha (HIFA) subunits leading to their proteasomal degradation in normoxia. Inactivation of VHL prevents HIFA degradation activating the heterodimeric transcription factors HIF1 and HIF2, and, thus, enables the activation of the cellular response to hypoxia even in normoxic conditions. We previously generated rod-specific *Vhl* knockout mice ($rod^{\varDelta \ Vhl}$) where chronic HIF1 (but not HIF2) activation led to a late-onset and age-dependent PR and RPE degeneration, mimicking aspects of retinal degenerative diseases associated with aging in humans [30, 31].

In this study, we addressed the relationship between energy metabolism and PR degeneration using chronic HIF activation as a model of age-related retinal degeneration. Using two-photon laser scanning microscopy (TPLSM) of genetically encoded biosensors, layer-specific proteomics and *in situ* enzymatic assays, we analyzed lactate and glucose dynamics at the cellular level, investigated oxidative phosphorylation pathway (OXPHOS) and the tricarboxylic acid (TCA) cycle, and addressed their contribution to PR survival.

## Results

### Chronic HIF activation in rods reduces outer segment length and increases lactate production

**Fig. 1:**
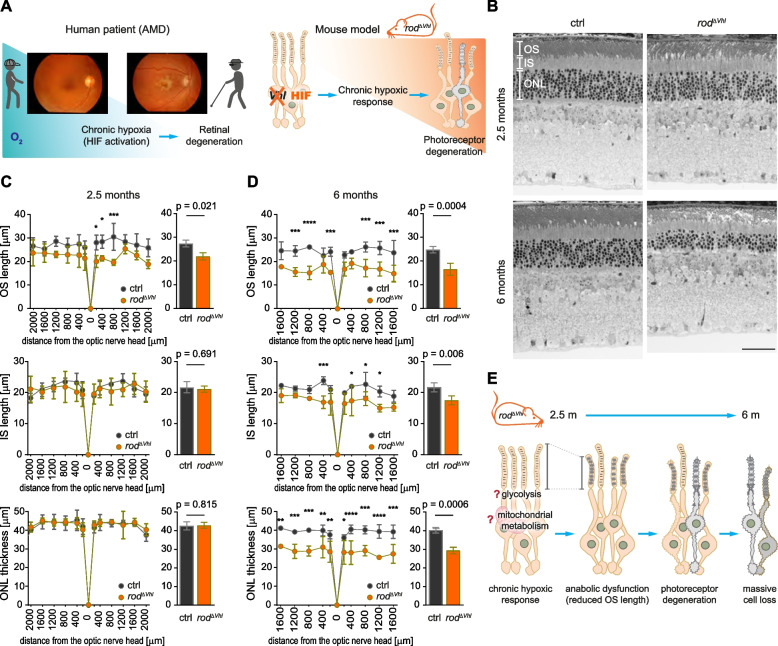
Reduced OS length in rods with chronically active HIFs. **a** Deletion of *Vhl* leads to chronic activation of HIF transcriptional factors and serves as a model for the aged human retina, in which reduced O_2_ delivery may cause tissue hypoxia contributing to development of AMD. **b** Representative micrographs of the retinal morphology of $rod^{\varDelta \ Vhl}$ and control (ctrl) mice at 2.5 and 6 months of age. Scale bar, 50 $\upmu$m. **c**,**d** Spidergrams (left) and bar graphs (right) of OS length (top), IS length (middle) and ONL thickness (bottom) in ctrl (black) and $rod^{\varDelta \ Vhl}$ (orange) mice at 2.5 (**c**, *N* = 4) and 6 months (**d**, *N* = 3 $rod^{\varDelta \ Vhl}$, *N* = 4 ctrl) of age. N: number of mice (one eye per mouse in the analysis). All measurements from the spidergrams are included in the bar graphs. Data represent mean ± SD. Statistics: two-way ANOVA and Bonferroni’s multiple comparison test (spidergrams) or nested *t*-test (bar graphs). *: *p* $\le$ 0.05. **: *p* $\le$ 0.01. ***: *p* $\le$ 0.001. ****: *p* $\le$ 0.0001. **e** Chronic HIF activation may affect anabolism through altered glycolysis and mitochondrial metabolism leading to reduced OS biogenesis preceding PR degeneration. OS: outer segments. IS: inner segments. ONL: outer nuclear layer

Age-related changes may impair oxygen flux from the choroid to the RPE and outer retina, generating a hypoxic environment in the retina. Hypoxia activates an intracellular molecular response that includes activation of HIF transcription factors and production of vascular endothelial growth factor (VEGF), the main driver of neovascularization in wet AMD [32]. However, chronic hypoxia not only drives neovascularization but also profoundly affects various cellular metabolic processes that may contribute to the development of dry AMD. To generate a mouse model that mimics the situation of the retina in the aged eye, we inactivated *Vhl* specifically in rods using the *Vhl*^*flox/flox*^;*OpsinCre* ($rod^{\varDelta \ Vhl}$) mice [30, 31] (Fig. 1a) . *Vhl* inactivation led to stabilization and chronic activation of HIF transcription factors in rods resulting in a late onset and slowly progressing HIF1-dependent PR degeneration [30, 31] (Fig. 1b).

To identify mechanisms leading to the degeneration in this disease model, we focused on early alterations. Already at 2.5 months of age, a time point before PR degeneration started, rod OS were significantly shorter in $rod^{\varDelta \ Vhl}$ mice while outer nuclear layer (ONL) thickness and IS length were similar to controls (Fig. 1c). OS shortening progressed with time and was accompanied by reduced IS length and ONL thickness at 6 months of age (Fig. 1d). We hypothesized that chronic HIF activation led to early anabolic deficits affecting OS biosynthesis preceding the onset of PR degeneration. Since anabolism is closely linked to energy metabolism, we focused on glycolysis and mitochondrial metabolism, including OXPHOS and the TCA cycle, to unravel the mechanistic basis of the anabolic deficit (Fig. 1e).

**Fig. 2:**
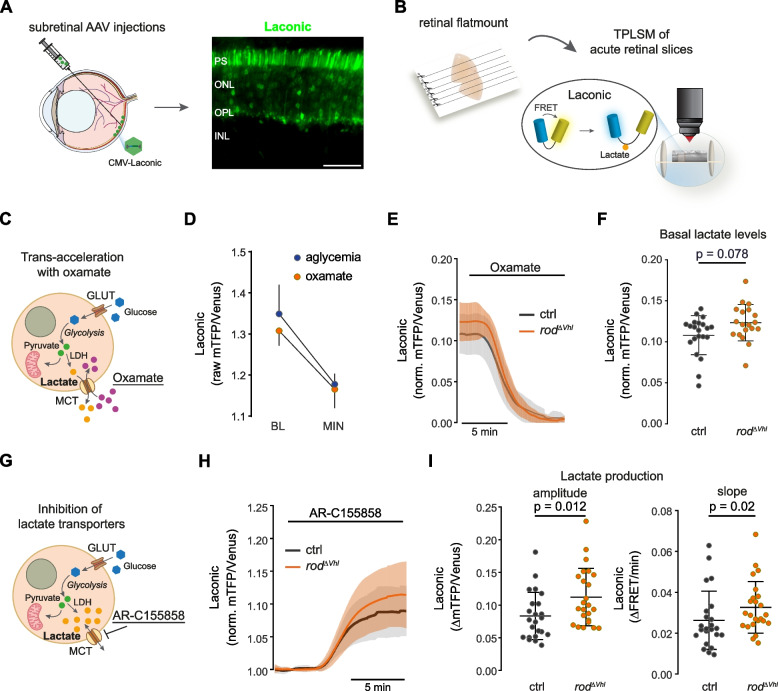
Increased lactate production in rods with chronically active HIFs. **a** Expression of the lactate sensor Laconic in photoreceptors after subretinal AAV application. Scale bar, 50 $\upmu$m. **b** Preparation of acute retinal slices from flat mounted half retinas and imaging of Laconic by TPLSM. **c** Schematic representation of monocarboxylate transporter (MCT) trans-acceleration with high extracellular levels of oxamate causing export of lactate. **d** Laconic raw FRET signal (individual values and means ± SD) in PRs at baseline (BL) and at minimum (MIN) obtained after exposure to oxamate (orange; 6 retinal slices from 2 mice) or to aglycemic medium (blue; 11 retinal slices from 4 mice). **e** Laconic traces (mean ± SD) in PRs of $rod^{\varDelta \ Vhl}$ (orange; 19 retinal slices from 10 mice) and ctrl mice (black; 21 retinal slices from 11 mice) during MCT trans-acceleration with oxamate. Traces were normalized to “zero” lactate (values at the end of the experiment). **f** Basal lactate levels (individual values and means ± SD) in $rod^{\varDelta \ Vhl}$ and ctrl PRs calculated from the laconic traces shown in (**e**). **g** Schematic representation of lactate transport inhibition by pharmacologically blocking MCT activity with AR-C155858. **h** Laconic traces (mean ± SD) in PRs of $rod^{\varDelta \ Vhl}$ (orange; 24 retinal slices from 11 mice) and ctrl mice (black; 23 retinal slices from 8 mice) during MCT block with AR-C155858. Traces were normalized to baseline (values before MCT inhibition). **i** Intracellular lactate accumulation (amplitude, left) and production rate (slope, right) calculated from the laconic traces shown in (**h**). Shown are individual values and means ± SD. Statistics: Mann-Whitney nonparametric test. PS: photoreceptor segments. ONL: outer nuclear layer. OPL: outer plexiform layer. INL: inner nuclear layer

To investigate glycolytic metabolites, we delivered FRET biosensors to PR using subretinal injections of adeno-associated viruses (AAV) and imaged the sensors in acute retinal slices by TPLSM (Fig. 2a,b). Since PRs are light sensitive cells and their metabolism may differ in light and darkness [7], we first investigated whether TPLSM activates phototransduction by monitoring Ca^2+^ levels during imaging using rod-specific expression of GCaMP6s (Fig. S1a,b). GCaMP6s is a genetically encoded single fluorophore calcium sensor consisting of a circularly permuted green fluorescent protein (cpGFP), the calcium-binding protein calmodulin (CaM), and the CaM-interacting M13 peptide [33]. Ca^2+^ binding to the CaM-M13 complex induces a conformational change in the protein leading to an increase in the brightness of cpGFP that is detectable by microscopy (Fig S1b).

TPLSM induced a drop in the fluorescence signal intensity in photoreceptor segments (PS) and synaptic terminals in the outer plexiform layer (OPL) of wild-type rods (Fig. S1c,d), indicating a decrease in intracellular Ca^2+^ concentrations during imaging. The stable GCaMP6s fluorescence signal in *Gnat1a*^*-/-*^ mice with their light-insensitive rods [34] (Fig. S1e) verified that the drop observed in wild-type mice was due to the activation of the phototransduction cascade by the laser light and/or by the fluorescence emitted from the sensor. Considering that the emission spectra of all used biosensors overlap, our imaging data for lactate and glucose relate to light-exposed rods.

We used the FRET biosensor Laconic [35] to analyze lactate, the end product of glycolysis. Laconic is a fusion protein composed of the lactate binding bacterial transcription regulator LldR and the FRET pair mTFP and Venus. Binding of lactate decreases FRET efficiency leading to reduced Venus signal (Fig. 2b). To compare basal lactate levels in rods of $rod^{\varDelta \ Vhl}$ and control mice, we depleted intracellular lactate by using the trans-acceleration property of the monocarboxylate transporters (MCTs) upon application of the non-metabolized MCT substrate oxamate (Fig. 2c) [36]. Due to the symporter type carrier characteristics of the MCTs, occupancy of the transporters in trans by oxamate induces import of oxamate into and extrusion of lactate out of the cell. Successful depletion of intracellular lactate by oxamate was confirmed by assessing lactate levels in aglycemic conditions, which reduced the Laconic FRET signal to a similar level (Fig. 2d). When normalized to this nominal zero lactate, there was no significant difference between rod lactate levels in $rod^{\varDelta \ Vhl}$ and control mice (Fig. 2e,f). To test whether chronic HIF activation affected lactate production rather than lactate levels, we inhibited MCT mediated lactate transport across the cellular membrane with AR-C155858 [37] (Fig. 2g). This resulted in an increase of intracellular lactate (Fig. 2h) demonstrating that rods are net lactate producers in the presence of glucose as the exclusive fuel. Higher amplitude and higher rate of lactate accumulation suggested that PRs of $rod^{\varDelta \ Vhl}$ mice are faster lactate producers than those of control mice (Fig. 2h,i).

**Fig. 3:**
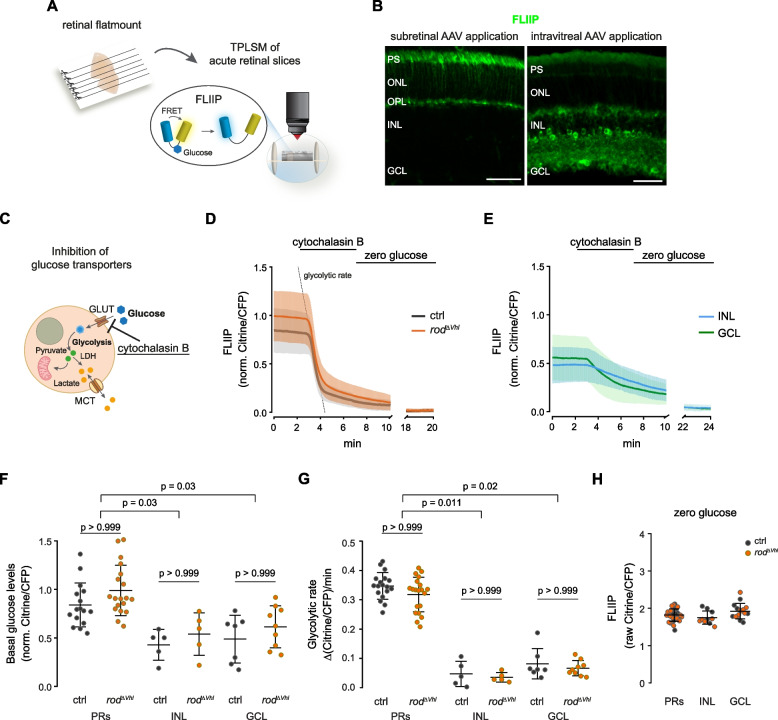
Glucose dynamics in photoreceptors with chronically active HIFs. **a** Preparation of acute retinal slices from flat mounted half retinas and imaging of the glucose sensor FLIIP by TPLSM. **b** Expression of FLIIP in PRs after subretinal AAV application (left) or in cells of the inner retina after intravitreal AAV application (right). Scale bar, 50 $\upmu$m. **c** Schematic representation of glucose transport inhibition by cytochalasin B allowing to monitor glycolytic rate through the hexokinases. **d** FLIIP traces (mean ± SD) in PRs from $rod^{\varDelta \ Vhl}$ (orange; 19 retinal slices from 5 mice) and ctrl mice (black, 16 retinal slices from 4 mice) during inhibition of glucose transport with cytochalasin B followed by aglycemia (zero glucose). Traces were normalized to zero glucose (values at the end of experiment). **e** FLIIP traces (mean ± SD) in cells of the INL (blue; 10 retinal flatmounts from 7 mice) and GCL (green; 16 retinal flatmounts from 7 mice) during inhibition of glucose transport with cytochalasin B followed by aglycemia (zero glucose). Traces were normalized to zero glucose (values at the end of experiment). **f** Basal glucose levels in PRs and cells of the INL and GCL in $rod^{\varDelta \ Vhl}$ and ctrl mice as calculated from the FLIIP traces shown in (**d**) and (**e**). Shown are individual values and means ± SD. **g** Glucose consumption rate in PRs and cells of the INL and GCL in $rod^{\varDelta \ Vhl}$ and ctrl mice as calculated from the FLIIP traces shown in (**d**) and (**e**). Shown are individual values and means ± SD. **h** Comparison of FLIIP raw FRET signals in PRs and cells of the INL and GCL in $rod^{\varDelta \ Vhl}$ (orange) and ctrl (black) mice at zero intracellular glucose. Statistics: Mann-Whitney nonparametric test for comparison of $rod^{\varDelta \ Vhl}$ and ctrl mice. Kruskal-Wallis nonparametric test and Dunnett’s multiple comparison test for comparison of PRs, GCL and INL cells. PS: photoreceptor segments. ONL: outer nuclear layer. OPL: outer plexiform layer. INL: inner nuclear layer. GCL: ganglion cell layer

Increased lactate production may be a consequence of increased glycolysis or decreased mitochondrial pyruvate consumption. We tested the first possibility by analysing intracellular glucose dynamics in retinal cells by TPLSM of the FRET glucose sensor FLIIP (Fig. 3a). FLIIP is composed of the glucose/galactose binding protein of *Escherichia coli* (MglB) and the FRET pair eCFP and Citrine and exhibits an increase in FRET efficiency upon glucose binding [38]. The sensor was delivered to PRs and to inner retinal cells by subretinal and intravitreal AAV injections, respectively (Fig. 3b). The rate of glycolysis at its entry point was estimated by pharmacological inhibition of glucose import using cytochalasin B, a competitive inhibitor of glucose transporters (Fig. 3c-e) [39–41]. Basal glucose levels were assessed by normalizing the FRET traces to nominal zero glucose using medium without glucose (aglycemia) at the end of the experiment (Fig. 3d,e). Surprisingly, baseline glucose levels and glucose consumption rate in PRs did not differ between $rod^{\varDelta \ Vhl}$ and control mice (Fig. 3f,g), suggesting that chronic HIF activation did not accelerate glycolysis in PRs and that increased intracellular lactate levels may have resulted from reduced mitochondrial pyruvate utilization. The very sharp drop of the FRET signal after blocking glucose import indicated that PRs have a remarkably high glucose consumption rate that was significantly higher than that observed in cells of the inner nuclear layer (INL) and ganglion cell layer (GCL) (Fig. 3d,e,g). Similarly, basal glucose levels were higher in PRs than in cells of the inner retina (Fig. 3d-f). Although FRET sensors may be affected by the cellular environment [42], raw FRET signals from FLIIP in aglycemic conditions were comparable between PRs, and cells of the INL and GCL (Fig. 3h), suggesting a similar sensor behavior in the different retinal cell types.

**Fig. 4:**
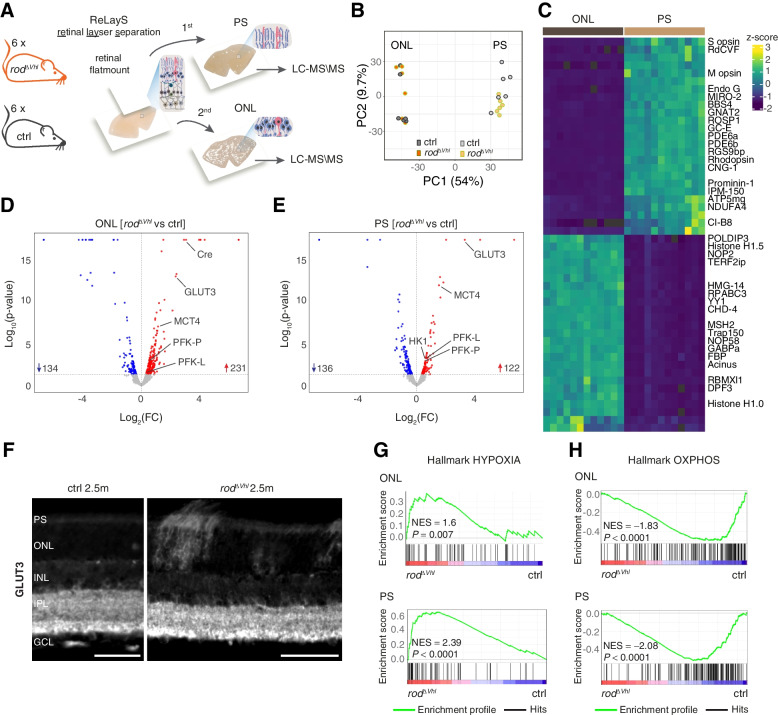
Proteomic landscape of $rod^{\varDelta \ Vhl}$ PS and ONL. **a** Photoreceptor segments (PS) and the outer nuclear layer (ONL) were isolated from six $rod^{\varDelta \ Vhl}$ and six ctrl mice and their proteomic landscape analysed by LC-MS/MS. **b** Principal component analyses of the PS and ONL proteomes from $rod^{\varDelta \ Vhl}$ and ctrl mice. **c** Heatmap of the top 25 up- and downregulated proteins in the ONL compared to the PS samples of all 12 mice. Proteins belonging to the phototransduction cascade, cilium, mitochondria or nucleus are indicated. **d**,**e** Volcano plots showing differentially up- (red) and downregulated (blue) proteins in the ONL (**d**) and PS (**e**) of $rod^{\varDelta \ Vhl}$ mice compared to ctrl. Total number of differentially regulated proteins as indicated. Threshhold: p $\le$ 0.05. **f** Representative immunofluorescence labeling for GLUT3 in $rod^{\varDelta \ Vhl}$ and ctrl retina. Scale bar, 50 $\upmu$m. *N* = 3 mice per genotype. **g**,**h** Gene set enrichment analyses using the MSigDB hallmark dataset. Enrichment of proteins of the hallmark HYPOXIA (**g**) and OXPHOS (**h**) in the ONL and PS of $rod^{\varDelta \ Vhl}$ and ctrl mice as indicated. The top of each panel shows the running enrichment score (green) for the gene set. The lower part of each panel shows the position of identified members of the gene set (black lines: hits, red to blue gradient: strength of enrichment in $rod^{\varDelta \ Vhl}$ and control (ctrl) mice) in the ranked list of proteins. For statistical analysis on proteomics data see Methods. NES: normalized enrichment score. PS: photoreceptor segments. ONL: outer nuclear layer. INL: inner nuclear layer. IPL: inner plexiform layer. GCL: ganglion cell layer

Together, our data showed that even though glucose dynamics were not altered, lactate production was enhanced in rods of $rod^{\varDelta \ Vhl}$ mice, suggesting that pyruvate is redirected to lactate upon chronic HIF activation. Since pyruvate is the main mitochondrial substrate and most of the mitochondria within PRs are localized in the IS, we established the retinal layer separation (ReLayS) method [43] and used proteomics (Fig. 4a) to investigate the protein landscapes in PS and soma (ONL) of $rod^{\varDelta \ Vhl}$ and control mice. Principal component analysis (PCA) of PS and ONL proteomes showed a strong separation between the two groups (Fig. 4b), indicating their markedly different protein compositions. The PS samples were strongly enriched in proteins belonging to the phototransduction cascade, the cilium and mitochondria, while many of the most abundant proteins in the ONL samples were nuclear proteins (Fig. 4c and Table S1) validating the separation procedure. Several proteins involved in glycolysis were significantly upregulated in the ONL (Fig. 4d and Table S2) and PS (Fig. 4e and Table S3) of $rod^{\varDelta \ Vhl}$ mice. Of particular interest were the increased levels of the glucose transporter GLUT3 (encoded by *Slc2a3*), since GLUT3 is not present in PRs under physiological conditions [44, 45]. This finding was verified by immunofluorescence studies showing a strong but patchy GLUT3 signal in the ONL and PS of $rod^{\varDelta \ Vhl}$ but not control mice (Fig. 4f), which resembled the expression of Cre in the ONL of $rod^{\varDelta \ Vhl}$ mice (Fig. S2). Importantly, we also detected increased levels of the lactate transporter MCT4 (encoded by *Slc16a3*) in both the ONL (Fig. 4d and Table S5) and PS (Fig. 4e and Table S3) of $rod^{\varDelta \ Vhl}$ mice. This is of significance since MCT4 is not blocked by AR-C155858 [37], the inhibitor used to determine lactate production by TPLSM (Fig. 2g-i). Thus, our imaging results may have even somewhat underestimated the magnitude of the lactate production in PRs of $rod^{\varDelta \ Vhl}$ mice, because lactate could still have been exported through the MCT4.

Finally, gene set enrichment analysis (GSEA) yielded an increased enrichment score of proteins belonging to the hallmark hypoxia and a reduced score of proteins involved in OXPHOS in both PS and ONL of $rod^{\varDelta \ Vhl}$ mice (Fig. 4g,h). This indicated that the chronic activation of HIFs in rods caused a proteomic landscape characteristic for hypoxia and suggested that this intracellular environment profoundly affected OXPHOS.

### Chronic HIF activation in rods reduces OXPHOS

**Fig. 5:**
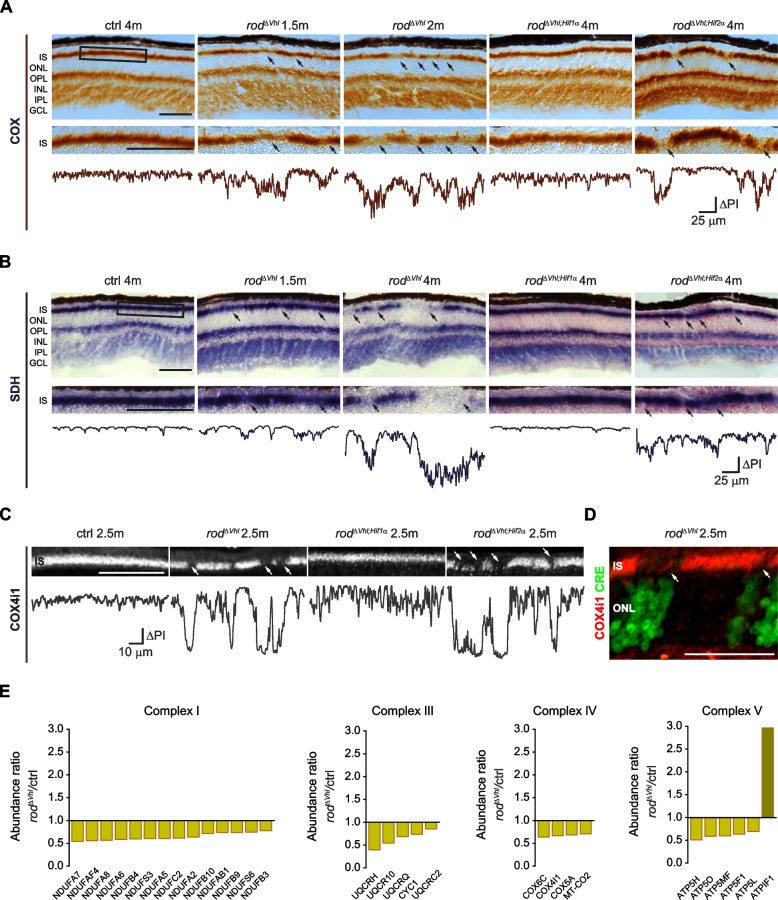
Dysregulation of OXPHOS in rods with chronically active HIFs. **a**,**b** Representative *in situ* enzymatic staining for COX (**a**) and SDH (**b**) activity in $rod^{\varDelta \ Vhl}$, $rod^{\varDelta \ Vhl;Hif1\alpha }$, $rod^{\varDelta \ Vhl;Hif2\alpha }$ and ctrl mice at time points as indicated. Shown are all retinal layers (top panels), a magnification of the photoreceptor segments (PS; boxed area, middle panels), and pixel intensity (PI) profiles through the PS (bottom panels). Black arrows indicate regions with reduced COX or SDH activity. *N* = 3 mice per genotype. **c** Representative immunofluorescence labeling for COX4i1 in the PS region of $rod^{\varDelta \ Vhl}$, $rod^{\varDelta \ Vhl;Hif1\alpha }$, $rod^{\varDelta \ Vhl;Hif2\alpha }$ and ctrl mice at 2.5 months of age (top panels). PI profiles through the PS (bottom panels). White arrows indicate regions with reduced COX4i1 labeling. *N* = 3 mice per genotype. **d** Representative immunofluorescence labeling for Cre (green) and COX4i1 (red) in the ONL and PS regions of $rod^{\varDelta \ Vhl}$ mice at 2.5 months of age. White arrows indicate regions with reduced COX4i1 labeling in the IS of Cre-positive cells. *N* = 3 mice. **e** Significantly downregulated OXPHOS complex subunits and assembly factors and upregulated complex V inhibitory factor ATPIF1 in the PS of $rod^{\varDelta \ Vhl}$ mice at 2.5 months of age. *N* = 6  mice per genotype. For statistical analysis on proteomics data see Methods. ONL: outer nuclear layer. OPL: outer plexiform layer. INL: inner nuclear layer. IPL: inner plexiform layer. GCL: ganglion cell layer. Scale bars, 50 $\upmu$m

Our TPLSM and proteomics data suggested a preferential conversion of pyruvate to lactate pointing to downregulation of OXPHOS in PRs of $rod^{\varDelta \ Vhl}$ mice. To address OXPHOS in PRs directly, we examined the activity of respiratory complexes IV (cytochrome c oxidase (COX)) and II (succinate dehydrogenase (SDH)) by *in situ* enzymatic assays. Prominent COX and SDH signals in the IS, the OPL, and the inner plexiform layer (IPL) of control mice indicated strong OXPHOS activity in the retinal regions known to be enriched in mitochondria (Fig. 5a,b). In $rod^{\varDelta \ Vhl}$ retinas, however, areas of reduced COX and SDH activities were evident in the IS already at 1.5 months of age, several weeks before the onset of degeneration. This phenotype deteriorated with age and depended on HIF1A but not HIF2A (black arrows in Fig. 5a,b) and, thus, on the same HIF isoform that drives retinal degeneration in $rod^{\varDelta \ Vhl}$ mice [31]. A HIF1 dependent impairment of COX was further evidenced by the absence of COX4i1 (a catalytic subunit of COX) in parts of the IS in $rod^{\varDelta \ Vhl}$ and $rod^{\varDelta \ Vhl;Hif2\alpha }$ (white arrows in Fig. 5c) but not $rod^{\varDelta \ Vhl;Hif1\alpha }$or control retinas. The patchy pattern of COX4i1 expression was very similar to the pattern of COX and SDH activity (Fig. 5a,b) and was likely due to the mosaic Cre expression in *OpsinCre* mice [30, 46] (Fig. S2b). Indeed, only Cre-positive areas of the ONL were associated with reduced COX4i1 levels in the adjacent IS (white arrows in Fig. 5d). It is unlikely that this effect was specific for the mitochondria in the rod IS, however, mitochondria localized in the photoreceptor synaptic terminals could not be assessed due to their close proximity to dendritic mitochondria belonging to 2^nd^ order neurons in the retina.

Since reduced activity of the OXPHOS complexes II and IV in rods of $rod^{\varDelta \ Vhl}$ mice corroborated the GSEA analyses of the layer-specific proteomic data (Fig. 4h), we further examined proteins directly related to OXPHOS, such as mitochondrial complex subunits and assembly factors. The mouse MitoCarta3.0 (http://www.broadinstitute.org/mitocarta) lists 165 such proteins, 81 of which were identified with high confidence in the PS of $rod^{\varDelta \ Vhl}$ and control mice. 28 of these proteins, belonging to complexes I, III, IV or V, including COX4i1, were significantly downregulated in $rod^{\varDelta \ Vhl}$ PS (Fig. 5e). The only upregulated OXPHOS-related protein was the ATPase inhibitory factor 1 (ATPIF1), suggesting complex V inhibition in rods of $rod^{\varDelta \ Vhl}$ mice. Interestingly, BNIP3 was detected in three of the six PS samples from $rod^{\varDelta \ Vhl}$ but in none of the control mice (Table S5). Although its presence in $rod^{\varDelta \ Vhl}$ was inconsistent, BNIP3 may be an interesting protein for further investigations due to its connection to cell death [47] and mitochondrial energetics [48]. Such future experiments will address the potential of BNIP3 to reduce rod survival in conditions of activated HIF proteins. Collectively, these data indicated that chronic HIF1 activation in rods caused OXPHOS dysregulation preceding the onset of cell degeneration.

### Chronic HIF activation in rods affects mitochondrial protein composition

**Fig. 6:**
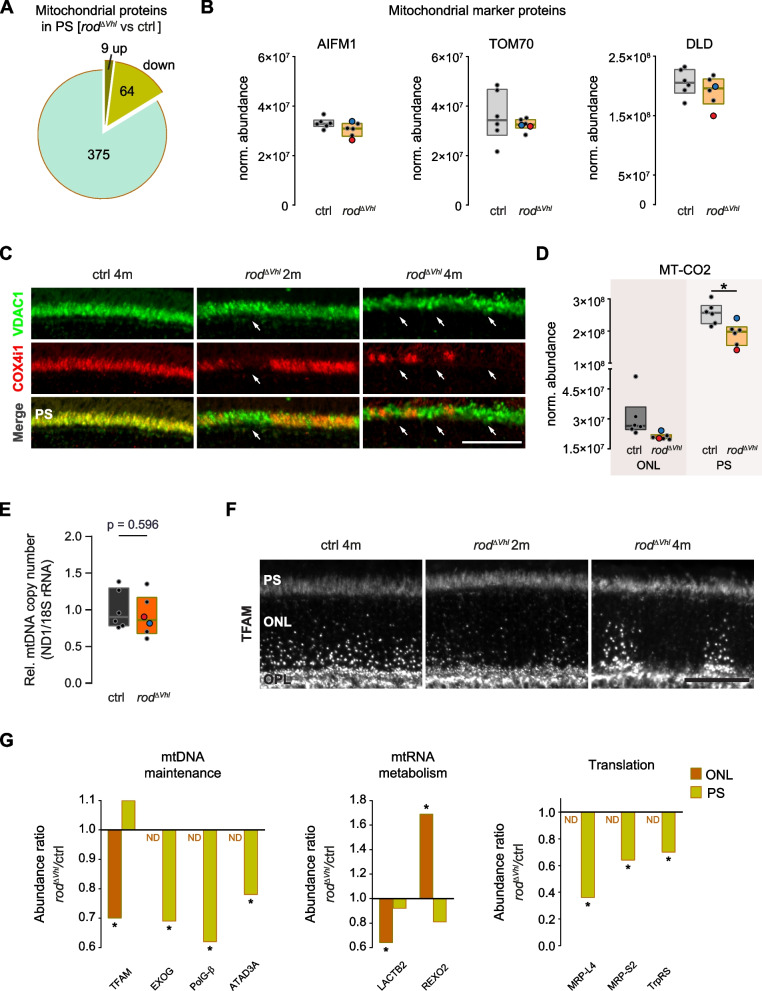
Mitochondria in rods with chronically active HIFs. **a** Pie chart of all identified and differentially regulated mitochondrial proteins in the photoreceptor segments (PS) of $rod^{\varDelta \ Vhl}$ mice at 2.5 months of age. *N* = 6 mice per genotype. **b** Protein levels of the mitochondrial markers AIFM1, TOM70, and DLD in PS of $rod^{\varDelta \ Vhl}$ mice and ctrl mice at 2.5 months of age. Shown are boxplots with the median and 25% and 75% percentiles of *N* = 6 mice per genotype. The samples with the highest (red) and lowest (blue) Cre levels are indicated. **c** Representative immunofluorescence labeling for VDAC1 (green, top panels) and COX4i1 (red, middle panels) in the PS region of $rod^{\varDelta \ Vhl}$ and ctrl mice at the indicated time points. Bottom panels: merge. White arrows: regions with reduced COX4i1 labeling. *N = 3* mice per genotype. **d** MT-CO2 levels in outer nuclear layer (ONL) and PS of $rod^{\varDelta \ Vhl}$ and ctrl mice at 2.5 months of age. Shown are boxplots with the median and 25% and 75% percentiles of *N* = 6 mice per genotype. The samples with the highest (red) and lowest (blue) Cre levels are indicated. **e** Relative quantification of mtDNA copy number in $rod^{\varDelta \ Vhl}$ and ctrl mice at 2.5 months of age. Shown are boxplots with the median and 25% and 75% percentiles of *N* = 6 mice per genotype. The samples with the highest (red) and lowest (blue) Cre levels are indicated. Statistics: Student’s *t*-test. **f** Representative immunofluorescence labeling for TFAM in the ONL and PS of $rod^{\varDelta \ Vhl}$ and ctrl mice at time points as indicated. *N = 3* mice per genotype. **g** Significantly regulated proteins involved in mtDNA maintenance (left), mtRNA metabolism (middle), and mitochondrial translation (right) in the PS and ONL of $rod^{\varDelta \ Vhl}$ mice at 2.5 months of age. *N* = 6 mice per genotype. ND, not detected. For statistical analysis on proteomics data see Methods. *: *p* $\le$ 0.05. OPL: outer plexiform layer. Scale bars, 50 $\upmu$m

Downregulation of OXPHOS-related proteins in PRs of $rod^{\varDelta \ Vhl}$ mice was unlikely due to loss of mitochondria. Only 14% of the mitochondrial proteins identified in the PS of $rod^{\varDelta \ Vhl}$ mice were significantly downregulated (Fig. 6a and Table S4) and well-known mitochondrial marker proteins, such as mitochondrial apoptosis-inducing factor 1 (AIFM1), mitochondrial import receptor subunit TOM70 (TOM70) and dihydrolipoamide dehydrogenase (DLD) had comparable levels in PS of $rod^{\varDelta \ Vhl}$ and control mice (Fig. 6b). Immunofluorescence labeling for voltage dependent anion channel 1 (VDAC1) also did not indicate loss of mitochondria in rods lacking COX4i1 in $rod^{\varDelta \ Vhl}$ retinas (white arrows point to areas with reduced COX4i1 staining in Fig. 6c). *Vdac1* expression was even significantly upregulated in the PS of $rod^{\varDelta \ Vhl}$ mice (Fig. S3a). To address a possible increase in transcriptional regulation of mitochondrial proteins, we assessed the relative expression levels of translocase of outer mitochondrial membrane 20 (*Tomm20*), another outer mitochondrial membrane protein. However, *Tomm20* expression was significantly downregulated in ONL and PS samples from $rod^{\varDelta \ Vhl}$ mice (Fig. S3a) suggesting that there was no general increase in transcription of mitochondrial genes. Together, these data indicate that chronic HIF activation in rods inhibited OXPHOS without affecting the overall mitochondrial biomass.

Although only one (cytochrome c oxidase subunit 2 (MT-CO2)) of the five mtDNA-encoded proteins identified in our proteomics data was downregulated in rods of $rod^{\varDelta \ Vhl}$ mice (Fig. 5e, Fig. 6d and Table S5), we examined whether OXPHOS downregulation was a result of compromised mtDNA integrity. Relative quantification of mtDNA copy number showed no difference between PRs of $rod^{\varDelta \ Vhl}$ and control mice (Fig. 6e), indicating that mtDNA integrity was not strongly affected by chronic HIF activation at 2.5 months of age.

Nevertheless, immunofluorescence labeling for the nuclear-encoded mitochondrial transcription factor A (TFAM) showed decreased immunoreactivity in patchy areas in the ONL of $rod^{\varDelta \ Vhl}$ mice as early as at 2 months of age, a phenotype that became even more pronounced with increasing age (Fig. 6f). Consistently, proteomics data showed a significant downregulation of TFAM and three other proteins involved in mtDNA maintenance (EXOG, PolG-$\upbeta$, ATAD3) in PRs of 2.5 months old $rod^{\varDelta \ Vhl}$ mice (Fig. 6g). In addition, two proteins involved in mtRNA metabolism (LACTB2 and REXO2) and three in mitochondrial translation (MRP-L4, MRP-S2, and TrpRS) were differentially and mostly downregulated in the ONL and PS of $rod^{\varDelta \ Vhl}$ mice (Fig. 6g). Similarly, expression of two mtDNA-encoded genes (*mt-Co2* and NADH-ubiquinone oxidoreductase chain 1 *(mt-Nd1)*) was significantly downregulated in ONL and PS samples of $rod^{\varDelta \ Vhl}$ mice (Fig. S3b). Together, our data suggest that chronic HIF1 activation in rods led to an early severe downregulation of OXPHOS-related proteins without a significant loss of mitochondria despite an affected mtDNA maintenance machinery. Furthermore, rods seemed to survive despite OXPHOS dysfunction, at least for some time.

### Inhibition of OXPHOS in rods does not lead to early anabolic deficiency

**Fig. 7:**
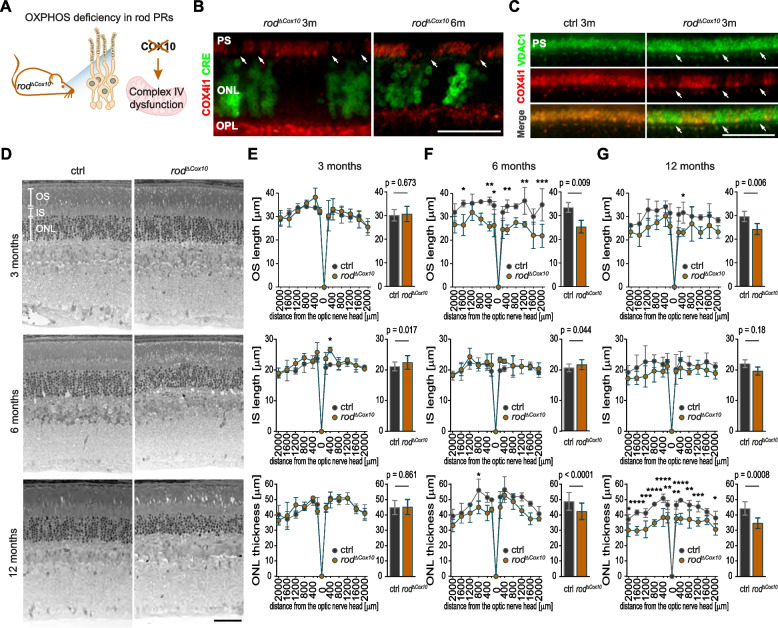
Rods survival in the absence of *Cox10*. **a** Mouse model for OXPHOS deficiency in rods ($rod^{\varDelta \ Cox10}$). *Cox10* deletion impairs assembly and function of the mitochondrial complex IV. **b** Representative immunofluorescence labeling for Cre (green) and COX4i1 (red) in the ONL and PS regions of $rod^{\varDelta \ Cox10}$ mice at time points as indicated. White arrows: regions with reduced COX4i1 labeling in the PS of Cre-positive cells. *N* = 3 mice per time point. **c** Representative immunofluorescence labeling for VDAC1 (green, top panels) and COX4i1 (red, middle panels) in the PS region of $rod^{\varDelta \ Cox10}$ and ctrl mice at 3 months of age. Bottom panels: merge. White arrows: regions with reduced COX4i1 labeling. *N* = 3 mice per genotype. **d** Representative micrographs of the retinal morphology of $rod^{\varDelta \ Cox10}$ and ctrl mice at time points as indicated. **e**-**g** Spidergrams (left) and bar graphs (right) of OS length (top), IS length (middle) and ONL thickness (bottom) in ctrl (black) and $rod^{\varDelta \ Cox10}$ (orange) mice at 3 (**e**, *N* = 3 mice per genotype), 6 (**f**, *N* = 3 mice per genotype), and 12 months (**g**, *N* = 4 mice per genotype) of age. All measurements from the spidergrams are included in the bar graphs. Data represent mean ± SD. Statistics: two-way ANOVA and Bonferroni’s multiple comparison test (spidergrams) or nested *t*-test (bar graphs). *: *p* $\le$ 0.05. **: *p* $\le$ 0.01. ***: *p* $\le$ 0.001. ****: *p* $\le$ 0.0001. PS: photoreceptor segments. OS: outer segments. IS: inner segments. ONL: outer nuclear layer. OPL: outer platform layer. Scale bars, 50 $\upmu$m

To further investigate the dependency of rod OS biogenesis and survival on OXPHOS, we generated $rod^{\varDelta \ Cox10}$ mice that have impaired OXPHOS activity due to compromised complex IV assembly [49] in rods (Fig. 7a). Similar to chronic HIF activation, *Cox10* deletion (indicated by the presence of Cre) led to the loss of the complex IV subunit COX4i1 (Fig. 7b) but not the outer mitochondrial membrane protein VDAC1 (Fig. 7c; white arrows point to areas with reduced COX4i1 staining) in rod IS. Furthermore, rods showed remarkable resilience against impaired OXPHOS activity and survived for several months (Fig. 7b,d-f) as indicated by the presence of Cre-positive rods in 6-month-old $rod^{\varDelta \ Cox10}$ mice (Fig. 7b). At this time point, ONL thickness was only moderately affected (Fig. 7d,f) and scotopic and photopic electroretinograms were in the normal range (Fig. S4). Significant loss of rods and reduced scotopic function were observed only at 12 months of age (Fig. 7d,g and Fig. S4). In strong contrast to $rod^{\varDelta \ Vhl}$ mice, OS length in $rod^{\varDelta \ Cox10}$ mice was not affected at the early age (Fig. 7d,e), indicating that OS biogenesis did not depend on OXPHOS activity.

### Chronic HIF activation in rods downregulates the citric acid cycle independently of OXPHOS

**Fig. 8:**
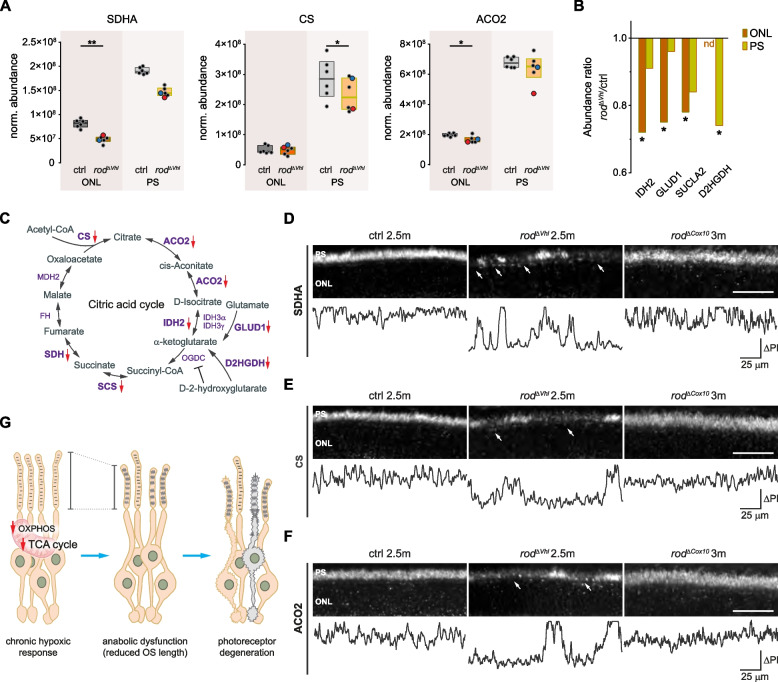
Dysregulation of the citric acid cycle in rods with chronically active HIFs. **a**,**b** Significantly downregulated citric acid cycle associated enzymes in photoreceptors segments (PS) or outer nuclear layer (ONL) of $rod^{\varDelta \ Vhl}$ mice at 2.5 months of age. Protein levels are shown as boxplots with the median and 25% and 75% percentiles (**a**) or bar graphs representing normalized abundance ratios of $rod^{\varDelta \ Vhl}$/ctrl (**b**). *N* = 6 mice per genotype. The samples with the highest (red) and lowest (blue) Cre levels are indicated. For statistical analysis on proteomics data see Methods. nd, not detected. *: *p* $\le$ 0.05. **: *p* $\le$ 0.01. **c** Representation of the citric acid cycle with red arrows marking significantly downregulated proteins in the PRs of $rod^{\varDelta \ Vhl}$ mice. **d**-**f** Representative immunofluorescence labeling for SDH subunit A (SDHA; **d**), citrate synthase (CS; **e**), and aconitate hydratase (ACO2; **f**) in the PS regions of $rod^{\varDelta \ Vhl}$, $rod^{\varDelta \ Cox10}$ and ctrl mice at time points as indicated (top panels). Pixel intensity (PI) profiles through the PS (bottom panels). White arrows: regions with reduced labeling. Scale bars, 50 $\upmu$m. *N* = 3 mice per genotype. **g** Dysregulation of OXPHOS and the TCA cycle in rods with chronic HIF activation is associated with impaired of OS biogenesis and leads to PRs degeneration

Since enzymatic staining showed a downregulation of SDH in $rod^{\varDelta \ Vhl}$ mice, an enzyme complex serving not only OXPHOS but also the TCA cycle, we hypothesised that chronic HIF activation affects the TCA cycle and, thus, generation of TCA metabolites important for anabolism and OS biogenesis. Detailed analysis of the proteomics data revealed a significant downregulation not only of SDH subunit A (SDHA) but also of six additional TCA associated enzymes in $rod^{\varDelta \ Vhl}$ mice (Fig. 8a-c). Immunofluorescence labeling for SDHA, citrate synthase (CS), and aconitase 2 (ACO2) validated the proteomics data and indicated that the TCA cycle was severely affected in $rod^{\varDelta \ Vhl}$ but not $rod^{\varDelta \ Cox10}$ mice (Fig. 8d-f). Together, these results suggest that chronic HIF activation concomitantly affected the TCA cycle and OXPHOS in rods, leading to anabolic dysfunction and ultimately to PR degeneration (Fig. 8g).

## Discussion

We investigated PR energy metabolism in a mouse model of slow progressing retinal degeneration [30, 31]. Our results show that PRs have a remarkably high glucose consumption rate, and that chronic HIF activation induces an increase in lactate production while severely affecting OXPHOS and the TCA cycle in rods. We also show that OXPHOS deficiency on its own is surprisingly well tolerated by rods, while additional impairment of the TCA cycle leads to an early dysregulation of rod OS biogenesis and retinal degeneration.

Since metabolism is fundamental factor for cell survival, tissue integrity and disease development, the investigation of glucose, the main metabolic precursor, becomes central for the understanding of pathophysiological processes. Using TPLSM and FRET biosensors, we provide, to our knowledge, the first data on lactate (Fig. 2) and glucose (Fig. 3) dynamics at cellular resolution in the intact retinal tissue. Results indicate that PRs are not only net lactate producers in the presence of glucose as the only fuel (Fig. 2h) but that they also have markedly higher basal glucose levels and glucose consumption rate than cells of the inner retina (Fig. 3). These results confirm the long-standing hypothesis of PRs being the main site of aerobic glycolysis in the retina [4, 12, 16, 18], a phenomenon that has been observed on tissue level already a century ago by Otto Warburg [9].

It is of interest that rods express GLUT3 upon chronic HIF activation (Fig. 4f), a glucose transporter with higher glucose affinity and greater transport capacity than GLUT1 [50]. Since GLUT3 is not present in rods under normoxic conditions [44, 45, 51], it is conceivable that rods may attempt to increase glucose uptake as a response to reduced OXPHOS activity during HIF activation. However, there was no measurable increase in basal glucose levels or glucose consumption rate in rods of $rod^{\varDelta \ Vhl}$ mice (Fig. 3). The absence of glycolysis stimulation in the face of OXPHOS failure was unexpected. One possibility is that in these cells the glycolytic rate is already near a maximum under normoxic conditions. Alternatively, chronic hypoxia may redirect anabolic branches like the pentose phosphate pathway towards the main glycolytic pathway for the production of ATP and lactate release, as observed in stressed neurons [52]. This redeployment of glucose would explain the combination of higher lactate production with conserved glucose consumption.

The increase of lactate production in rods upon chronic HIF activation (Fig. 2i) is in accordance to previous studies reporting oxygen level dependent lactate production in isolated retinas [53]. Notably, our data rather underestimated the magnitude of this increase for two reasons: First, the mosaic Cre expression (Fig. S2b) inevitably led to the inclusion of both *Vhl* knockout and wild type cells in our TPLSM measurements. Second, AR-C155858 potently inhibits MCT1 and MCT2 but not MCT4 [37] that is even significantly upregulated in the ONL and PS of $rod^{\varDelta \ Vhl}$ mice (Fig. 4d,e) and, thus, lactate could be exported from the cell through the high affinity MCT4 [54], escaping detection by the biosensor.

Since the glycolytic flux through the hexokinases was not altered, increased lactate production in rods of $rod^{\varDelta \ Vhl}$ mice was likely the result of a reduced pyruvate utilization by the mitochondria. In addition, feedback mechanisms and allosteric regulation of TCA cycle enzymes [55, 56] may also have affected function of mitochondrial metabolic pathways and thus contributed to the effect. Proteomics data from isolated PS and ONL (Fig. 4h, Fig. 5e, Fig. 8a,b) as well as results from enzymatic *in situ* experiments (Fig. 5a,b) and immunofluorescence studies (Fig. 5e, Fig. 8d-f) showed that chronic HIF1 (but not HIF2) activation in rods severely downregulated protein levels of several key enzymes involved not only in OXPHOS but also in the TCA cycle (Fig. 5, Fig. 8). Despite the downregulation of these proteins and the severely reduced activity of SDH and COX in photoreceptor segments, mitochondria remained present (Fig. 6c) and photoreceptors survived for several weeks (Fig. 1b,c). Survival of rods with OXPHOS deficiency was further corroborated in $rod^{\varDelta \ Cox10}$ mice, suggesting that the high glucose consumption rate of rods may generate sufficient energy for the cells to function and survive in such conditions. This extends the list of cells (including astrocytes [57], oligodendrocytes [58], brain neurons [59, 60], and skeletal muscle cells [49]) that are reported to survive as glycolytic cells in the absence of *Cox10*. Additionally, it has been suggested that cells in the retina can also use fumarate as electron acceptor instead of oxygen by reversing the succinate dehydrogenase reaction [61]. The resulting succinate can then be transported to the neighbouring RPE, oxidised to malate, and shuttled back to the retina. The existence of such malate-succinate shuttle could also help to explain the resilience of COX deficient rod PRs. Although PRs eventually die in both $rod^{\varDelta \ Vhl}$ (Fig. 1b-d) and $rod^{\varDelta \ Cox10}$ mice (Fig. 7d-g), the degenerative phenotype was more severe and appeared earlier in $rod^{\varDelta \ Vhl}$ mice. For survival, rods may thus not so much depend on OXPHOS but more heavily so on functional TCA cycle that is seemingly unaffected in $rod^{\varDelta \ Cox10}$ but not $rod^{\varDelta \ Vhl}$ mice. This underlines the significance of the TCA cycle with its pivotal role in anabolic and homeostatic processes that are essential, among others, for OS biogenesis. Reduced TCA cycle activity as well as shorter rod OS length have been reported in mouse models of retinitis pigmentosa (RP), an inherited retinal degenerative disorder with high genetic heterogeneity [62–65]. Shorter rod OS length was also described in RP patients [66, 67] as well as in the context of hypoxia in mice [68] and human [69]. Thus, TCA cycle dysregulation might represent a common molecular feature preceding PR degeneration. As suggested by others [64], supporting the TCA cycle may thus constitute a promising strategy to improve PR survival in a variety of patients.

## Conclusions

Our study demonstrates the consequences of an activated hypoxic response for glucose flux and lactate levels in rods and highlights the importance of the TCA cycle for rod survival. Despite the need for high ATP levels for maintenance of the visual function [7], rods survive in the absence of OXPHOS, indicating that they can satisfy their energy needs by alternative pathways such as aerobic glycolysis and/or beta-oxidation of fatty acids. In fact, it has been proposed that only about 20% of glucose is completely oxidized in the outer retina while the rest is used for lactate production [4] and that the long-chain fatty acid palmitate may serve as a fuel for mitochondrial respiration in the isolated retina [70, 71]. However, no sufficiently effective alternative pathway to the TCA cycle may exist to provide satisfactory amounts of intermediate metabolites needed for the constant biosynthesis of complex cellular structures such as the outer segments of PRs.

## Methods

### Animals

All experimental procedures were approved by the local veterinary authorities, conforming to the guidelines of the Swiss Animal Protection Law, Veterinary Office, Canton Zurich (Act of Animal Protection 16 December 2005 and Animal Protection Ordinance 23 April 2008) and were performed in accordance with the respective national, federal and institutional regulations. Mice were maintained as breeding colonies at the Laboratory Animal Services Center of the University of Zurich in a 14h:10h light-dark cycle. Mice had access to food and water *ad libitum*. The average light intensity at cage levels was 60-150 lux, depending on the position in the rack.

*Vhl*^*flox/flox*^ [72], *Hif1*$\alpha$ ^*flox/flox*^ [73], *Hif2*$\alpha$ ^*flox/flox*^ [74], *Cox10*^*flox/flox*^ [49], and *OpsinCre* (LMOPC1 [46]) mice were intercrossed to obtain $rod^{\varDelta \ Vhl}$ (*Vhl*^*flox/flox*^;*OpsinCre*), $rod^{\varDelta \ Vhl;Hif1\alpha }$(*Vhl*^*flox/flox*^;*Hif1*$\alpha$ ^*flox/flox*^;*OpsinCre*), $rod^{\varDelta \ Vhl;Hif2\alpha }$ (*Vhl*^*flox/flox*^;*Hif2*$\alpha$ ^*flox/flox*^; *OpsinCre*), and $rod^{\varDelta \ Cox10}$ (*Cox10*^*flox/flox*^;*OpsinCre*) mice. All breeding pairs were heterozygous for *OpsinCre* and littermates without *OpsinCre* served as controls. Rod-specific Cre expression in *OpsinCre* mice starts around postnatal day 7 and increases up to 6 weeks of age [46]. Homozygous rod transducin $\alpha$-subunit knockout mice [34] (*Gnat1a*^*-/-*^) were used as controls in TPLSM calcium imaging. All mice were homozygous for the *Rpe65*_*450Leu*_ variant [75, 76]. For tissue sample preparations, mice were euthanized with CO_2_ followed by decapitation.

### Retinal morphology analysis

Eyes were enucleated, fixed in glutaraldehyde (2.5% in cacodylate buffer) for 12-24 h at 4$^{\circ }$C, trimmed, post-fixed in 1% osmium tetroxide, and embedded in Epon 812 as described [77]. Retinal cross-sections of 0.5 $\upmu$m were cut through the optic nerve head, stained with toluidine blue, and analyzed by light microscopy (Zeiss, Axioplan). OS and IS length, and the thickness of the ONL were measured at indicated distances from the optic nerve head using the Adobe Photoshop CS6 ruler tool (Adobe). Mann-Whitney nonparametric test was used to compare the overall OS and IS length, as well as ONL thickness of $rod^{\varDelta \ Vhl}$ and $rod^{\varDelta \ Cox10}$ mice to their respective controls. *P*-values < 0.05 were considered to show significant differences.

### Cloning and virus production

The GCaMP6s gene [78] was cloned into an AAV plasmid backbone containing a rod PR-specific mouse opsin promoter (mOP) (a gift from Sanford L. Boye) [79]. The laconic AAV plasmid was a gift from Luis Felipe Barros (Addgene plasmid #44238) [35]. The FLIIP AAV plasmid [38] was codon-diversified to avoid recombination during AAV production [80, 81]. All AAVs were produced by the Viral Vector Facility of the Neuroscience Center Zurich and packaged in the AAV2(QuadYF+TV; 7m8)/2 capsid [82].

### Intraocular injections of AAVs carrying fluorescent sensors

For intraocular AAV injections, the pupils of mice were dilated with Cyclogyl 1% (Alcon Pharmaceuticals) and Neosynephrine 5% (Ursapharm Schweiz). Mice were anesthetized by a subcutaneous injection of ketamine (85 mg/kg, Parke-Davis) and xylazine (10 mg/kg, Bayer AG). Viscotears (Bausch & Lomb Swiss AG) were applied to keep the eyes moist. 1 x 10^9^ total viral genome in 1 $\upmu$l total volume were injected either intravitreally or subretinally to transduce the inner or the outer retinal cells, respectively. Anesthesia was reversed by a subcutaneous injection of antisedan (2 mg/kg, Atipazemole). Two to three weeks after injection, mice were subjected to fluorescent funduscopy and OCT to test for transgene expression and detect potential injection-inflicted tissue damage including bleeding or persistent retinal detachment. Such eyes were excluded from the study. $rod^{\varDelta \ Vhl}$ and ctrl mice were injected at 5-6 weeks of age and TPLSM experiments was performed at 10-14 weeks of age.

### Acute retinal preparations for TPLSM *ex vivo* imaging

Mice were housed in a facility with a reversed 12/12 h light/dark cycle for at least 5 days before the experiments. Prior to dissection, mice were dark adapted for at least 2 h, and all steps of the dissection procedure were carried out in dim red light. Retinas were rapidly dissected through a slit in the cornea and placed in freshly prepared artificial cerebrospinal fluid (ACSF) containing: 126 mM NaCl, 3 mM KCl, 2 mM CaCl_2_, 1.25 mM NaH_2_PO_4_, 26 mM NaHCO_3_, 2 mM MgSO_4_, 6 mM D-glucose), bubbled with oxycarbon (95% O2/ 5% CO2, PanGas, 820, and kept at RT. PRs were imaged in the cross-section of acute retinal slices as previously described [83]. In brief, half retinas were flatmounted with the PRs side facing up on a nitrocellulose filter membrane and cut in 250 $\upmu$m-thick slices using a razor blade attached to a custom-made tissue chopper. For imaging inner retinal cells, flatmounted retinas with the ganglion cells facing up were used without slicing the tissue. The tissue was left to rest while slowly warming the ACSF to 35$^{\circ }$C for 30 min before imaging.

### Two-photon excitation laser scanning microscopy

A custom-made two-photon laser scanning microscope equipped with a two-photon laser with < 120 fs temporal pulse width (InSight DeepSee Dual; Spectra-Physics) and a 16x water immersion objective (Nikon N16XLWD-PF, 0.8 NA, 3 mm WD) was used for image acquisition [84]. Excitation and emission beam paths were separated by a dichroic mirror (F73-825; AHF Analysentechnik). Dichroic mirrors at 506nm (F38-506; AHF Analysentechnik) and 560 nm (F38-560; AHF Analysentechnik) further separated the emission light into colored components, that were then focused on GaAsP photomultipliers (H10770PA-40sel; Hamamatsu Photonics) equipped with filters for cyan (475/50; AHF Analysentechnik), and green/yellow (542/50; AHF Analysentechnik) wavelenghts. ScanImage 3.8 [85] and custom-written LabVIEW software (Version 2012; National Instruments) were used for image control and data acquisition.

For imaging, constantly oxygenated ACSF was gravity-fed into the recording chamber at a flow rate of $\approx$ 2 ml/min and temperature was maintained at 35$^{\circ }$C using a temperature controller (V TC05, Luigs & Neumann GmbH) with an in-bath temperature sensor. All solutions used during imaging were infused using the same gravity flow system and osmolarity was adjusted at $\approx$ 300 mOsm. Data was acquired from acute retinal slices at 50-70 $\upmu$m below the cross-section surface, from cells in the GCL at 0-10 $\upmu$m and from cells in the INL at 50-60 $\upmu$m below the surface.

#### Calcium imaging parameters and analysis

GCaMP6s was excited at 940 nm wavelength and images were acquired at 11.84 Hz with a 128x256-pixel resolution. Analyses were performed with ImageJ (National Institutes of Health) and regions of interest were selected manually. Prism software (GraphPad) was used for data visualization.

#### Lactate and glucose imaging parameters and analysis

Laconic and FLIIP were excited at 870 nm wavelength and images were acquired at 5.94 Hz with a 256x256-pixel resolution. ACSF containing 20 mM oxamate was used in the trans-acceleration imaging protocol. ACSF containing 5 $\upmu$M AR-C155858 (BioTechne Tocris) was used in the lactate transport inhibition imaging protocol. ACSF containing 20 $\upmu$M cytochalasin B (BioTechne Tocris, 5474) was used in the glucose transport inhibition imaging protocol. ACSF containing 0 mM glucose was used in the aglycemia imaging protocol.

Whole-frame image analysis was carried out using custom-made code for MATLAB 2017b (MathWorks) and ImageJ (National Institutes of Health). For each experiment, images were aligned using a 2D convolution engine to account for xy drift in time. To optimize signal to noise ratio, time smoothing of 11 frames for Laconic and 5 frames for FLIIP was applied, as well as automatic thresholding using the Li’s minimum cross entropy method [86]. Ratiometric data were visualized with Prism software (GraphPad). Mann-Whitney nonparametric test was used to compare $rod^{\varDelta \ Vhl}$ and ctrl mice. Kuskal-Wallis nonparametric test and Dunnett’s multiple comparison test were used to compare PRs, GCL and INL cells. Because AAVs were injected subretinally and biosensor expression was CMV driven, both rod and cone PRs may have been transduced. However, given that rods outnumber cone PRs by a factor of 30 in the mouse retina, all imaging data represent primarily rods.

### Label-free quantitative proteome analysis

#### Sample preparation with the ReLayS method

Samples were prepared using the ReLayS method [43]. In brief, retinas from 2.5-months-old $rod^{\varDelta \ Vhl}$ (*N* = 6) and control (*N* = 6) mice were rapidly dissected through a slit in the cornea and placed in PBS on ice. Vitreous was carefully removed using a pair of forceps and retinas were halved through the optic nerve head. The half-retinas were flattened on a small piece of nitrocellulose filter membrane with the photoreceptor side facing up and frozen on a metal platform placed on dry ice. Frozen retinal flatmounts were stored at -80$^{\circ }$C until further use. PS and ONL samples were consecutively separated from frozen half-retinal flatmounts using the adherence of the cellular structures to a nitrocellulose filter membrane placed on top of the photoreceptors or the ONL, respectively. The PS and ONL membranes were frozen on a metal platform placed on dry ice and transferred to 1.5 ml Protein LoBind$^{\circledR }$ Tubes (Eppendorf; 0030108116). For protein isolation, samples were homogenized by sonication with an ultrasonic homogenizer in ice-cold Tris-HCl (100 mM, pH 8.0) containing protease inhibitors (Sigma-Aldrich, P2714). After sonication, 10% SDS (final concentration 1% SDS in Tris-HCl, pH 8.0) was added and samples were incubated at 75$^{\circ }$C for 10 min. Protein samples were stored at -20$^{\circ }$C until further use.

#### Mass spectrometry

The mass spectrometry proteomic data have been deposited to the ProteomeXchange Consortium via the PRIDE [87] partner repository with the dataset identifier PXD034057.

From each sample, total proteins were proteolyzed with LysC (Wako Chemicals, Neuss, Germany) and trypsin (Promega) using a suspension trapping protocol (S-Trap, Protifi) to remove SDS according to the manufacturer’s instructions. Briefly, samples were reduced and carbamidomethylated, followed by acidification with phosphoric acid and addition of methanol to a final concentration of >70% before loading to the trap columns. Proteins were washed while trapped on column, then digested on column with LysC (2 hours at RT) followed by trypsin (overnight, at 37$^{\circ }$C). Peptides were collected by centrifugation and acidified. Eluted peptides were analyzed on a Q Exactive HF mass spectrometer (Thermo Fisher Scientific) in the data dependent mode. Approximately 0.5 $\upmu$g peptides per sample were automatically loaded to the online coupled ultra-high-performance liquid chromatography (UHPLC) system (Ultimate 3000, Thermo Fisher Scientific). A nano trap column was used (300-$\upmu$m ID X 5mm, packed with Acclaim PepMap100 C18, 5 $\upmu$m, 100 Å; LC Packings) before separation by reversed phase chromatography (Acquity UHPLC M-Class HSS T3 Column 75 $\upmu$m ID X 250 mm, 1.8 $\upmu$m; Waters) at 40$^{\circ }$C. Peptides were eluted from the column at 250 nL/min using increasing ACN concentrations (in 0.1% formic acid) from 3% to 41% over a linear 95-min gradient. MS spectra were recorded at a resolution of 60 000 with an AGC target of 3 $\times$ 106 and a maximum injection time of 50 ms from 300 to 1’500 m/z. From the MS scan, the 10 most abundant peptide ions were selected for fragmentation via HCD with a normalized collision energy of 28, an isolation window of 1.6 m/z, and a dynamic exclusion of 30 s. MS/MS spectra were recorded at a resolution of 15’000 with an AGC target of 105 and a maximum injection time of 50 ms. Unassigned charges and charges of +1 and above +8 were excluded from precursor selection.

#### Data processing - protein identification and label-free quantification

Proteome Discoverer 2.4 software (Thermo Fisher Scientific; version 2.4.1.15) was used for peptide and protein identification via a database search (Sequest HT search engine) against SwissProt mouse database (release 2020_02, 17061 sequences), considering full tryptic specificity, allowing for up to two missed tryptic cleavage sites, precursor mass tolerance 10 ppm, and fragment mass tolerance 0.02 Da. Carbamidomethylation of Cys was set as a static modification. Dynamic modifications included deamidation of Asn and Gln, oxidation of Met; and a combination of Met loss with acetylation on protein N-terminus. Percolator [88] was used for validating peptide spectrum matches and peptides, accepting only the top-scoring hit for each spectrum, and satisfying the cut-off values for FDR < 1%, and posterior error probability < 0.01. The final list of proteins complied with the strict parsimony principle.

The quantification of proteins, after precursor recalibration, was based on abundance values (intensity) for unique peptides. Abundance values were normalized to the total peptide amount to account for sample load errors. The protein abundances were calculated by summing the abundance values for admissible peptides. The final protein ratio was calculated using median abundance values of six replicate analyses each. The statistical significance of the ratio change was ascertained employing the approach described in [89], which is based on the presumption that we look for expression changes for proteins that are just a few in comparison to the number of total proteins being quantified. The quantification variability of the non-changing “background” proteins can be used to infer which proteins change their expression in a statistically significant manner.

#### Data analysis and visualization

PCA was performed on normalized abundance protein levels using ClustVis [90] (http://biit.cs.ut.ee/clustvis/). Heatmap of the top 25 proteins in ONL and PS was generated from the scaled normalized abundance values using the ComplexHeatmap package + [91] and the viridis color palette with the R package circlize [92]. The volcano plots were generated using VolcaNoseR [93] (https://huygens.science.uva.nl/VolcaNoseR2/). GSEA was performed using GSEA (Version 4.1.0) software provided by Broad Institute of Massachusetts Institute of Technology and Harvard University [94, 95]. Analysis was conducted on a pre-ranked protein list based on their relative abundance in $rod^{\varDelta \ Vhl}$ against control samples for the PS and ONL, respectively. The hallmark gene set from Molecular Signatures Database [96, 97] (MSigDB, Version 7.2) was used for comparison. Normalized protein abundances and abundance ratios were visualized with Prism software (GraphPad).

### mtDNA copy number assay

PS and ONL samples were prepared from 2.5-months-old $rod^{\varDelta \ Vhl}$ (*N* = 6) and control (*N* = 6) mice using the ReLayS method [43] as described above. Tissue was lysed by proteinase K (Sigma-Aldrich, 03115879001) treatment at 56$^{\circ }$C for 45 min with shacking at 300 rpm and RNase A (100 mg/ml; Thermo Fisher Scientific, 12091021) was added to the samples. DNA isolation was performed with QIAamp DNA Blood Mini Kit (Qiagen, 51104) according to manufacturer instructions. DNA from PS and ONL samples was pooled together to obtain DNA samples from photoreceptors. Relative quantification of mtDNA levels was determined by the ratio of the mitochondrial ND1 (*mt-Nd1*) gene to the nuclear-encoded 18S rRNA gene using real-time PCR. 16 ng DNA was used as template and the genes of interest amplified using the PowerUp SYBR Green Master Mix (ThermoFisher Scientific) in the ABI QuantStudio 3 system (ThermoFisher Scientific) and specific primer pairs: ND1 fwd 5’-3’: CTAGCAGAAACAAACCGGGC and ND1 rev 5’-3’: CCGGCTGCGTATTCTACGTT; 18S rRNA fwd 5’-3’: CGCGGTTCTATTTTGTTGGT and 18S rRNA rev 5’-3’: AGTCGGCATCGTTTATGGTC. Data analysis was carried out using the 2$^{\wedge }$-ddCt method [98].

### Immunohistochemistry

Eyes were marked at the nasal limbus, enucleated, and fixed in 4% PFA in PBS for a total of 4h at 4$^{\circ }$C. For cryosectioning, eyes were placed in 30% sucrose in PBS for 2h at 4$^{\circ }$C and embedded with tissue freezing medium (Leica Biosystems, 81-0771-00). 12-$\upmu$m-thick sections were prepared using a Leica cryostat (Biosystems Switzerland AG, CM1860). For paraffin sections, PFA fixed eyes were dehydrated in a series of ethanol solutions of increasing concentrations up to 100% and immersed in xylene prior to paraffin embedding. 5-$\upmu$m-thick sections were prepared using a Zeiss microtome (Microm HM 440E). Paraffin sections were deparaffinized, rehydrated, and epitopes were unmasked by heat-induced antigen retrieval at pH 6. For immunofluorescence staining, paraffin- and cryosections were blocked with PBS containing 3% normal goat serum and 1% Triton X-100. The following primary antibodies were incubated overnight at 4$^{\circ }$C: rabbit anti-GLUT3 (1:250; Sigma-Aldrich, ab1344), mouse anti-COX4i1 (1:100; Abcam, ab14744), rabbit anti-CRE (1:300; Merck Millipore, 69050-3), rabbit anti-VDAC1 (1:250; Abcam, ab15895), rabbit anti-TFAM (1:250; Abcam, ab131607), rabbit anti-CS (1:250; GeneTex, GTX110624), rabbit anti-ACO2 (1:250; Cell Signaling Technology, 6571), rabbit anti-SDHA (1:250; Cell Signaling Technology, 11998) or rabbit anti-cARR (1:1000; Merck Millipore Chemicals, AB15282). Secondary antibodies (Alexa Fluor 488- or 568-conjugated, 1:500) were applied for 1 h at RT. Fluorescence images were acquired with a Zeiss microscope (Axioplan 2, Zeiss), processed, and analyzed with ImageJ (National Institutes of Health). Pixel intensity profiles through the PS were obtained using the profile plots tool controlled by the line tool in ImageJ.

### COX and SDH enzymatic histochemistry

Eyes were marked at the nasal limbus, enucleated, briefly washed in ice cold PBS, and immediately embedded in tissue freezing medium (Leica Biosystems, 81-0771-00). 12-$\upmu$m-thick sections were prepared using a Leica cryostat. For COX enzymatic histochemistry, sections were incubated with COX detection solution (1.6 mM DAB, 1.25 mg/ml cytochrom C, 10 $\upmu$l catalase in PBS, pH 7.0) for 45 min at 37$^{\circ }$C. For SDH enzymatic histochemistry, sections were incubated with SDH detection solution (1.55 mM nitrotetrazolium blue chloride, 0.13 mM sodium succinate, 0.2 mM phenazine methosulfate, 0.1 mM sodium azide in PBS, pH 7.0) for 25 minutes at 37$^{\circ }$C. Sections were dehydrated in a series of ethanol solutions of increasing concentrations up to 100% and immersed in xylene. Coverslips were mounted with Cytoseal (Thermo Scientific, 8312-4). Bright-field microscopy images were acquired with a Zeiss microscope (Axioplan 2), processed and analyzed with ImageJ (National Institutes of Health). Pixel intensity profiles through the PS were obtained using the profile plots tool controlled by the line tool in ImageJ.

### Scotopic and photopic electroretinography (ERG)

Mice were processed for ERG as described [99]. Briefly, after overnight dark-adaptation and pupil dilation with 1% Cyclogyl (Alcon) and 5% Neosynephrin-POS, mice were anesthetized with ketamin (85 mg/kg) and xylazine (10 mg/kg). Electroretinograms were recorded simultaneously from both eyes using a Diagnosys Celeris rodent ERG device (Diagnosys). Ten flash intensities ranging from 8x10^-6^ cd*s/m^2^ to 3 cd*s/m^2^ and six flash intensities ranging from 1 cd*s/m^2^ to 200 cd*s/m^2^ were used for dark- (scotopic) and light-adapted (photopic) single-flash intensity ERG series, respectively. Five sweeps per intensity were averaged for the scotopic and ten sweeps per intensity for the photopic ERGs. The standard rod-suppressive background light (30 cd/m^2^) was used prior (5 min) and during recordings in photopic conditions. Statistical analysis was performed using two-way ANOVA and Bonferroni’s multiple comparison test (spidergrams) or nested *t*-test (bar graphs) with Prism software (GraphPad).

### Semi-quantitative real-time PCR

PS and ONL samples were prepared from 2.5-months-old $rod^{\varDelta \ Vhl}$ (*N* = 6) and control (*N* = 6) mice using the ReLayS method [43] as described above. Total RNA was isolated from the tissue on the membrane with an RNA isolation kit (Thermo Fisher PicoPure RNA Isolation Kit, KIT0204) including an on-column DNaseI treatment. cDNA synthesis was carried out with oligo-(d)T primers and M-MLV reverse transcriptase (Promega). For semi-quantitative real-time PCR, 10 ng cDNA was amplified using the PowerUp SYBR Green Master Mix (Thermo Fisher Scientific) and specific primer pairs (*Actb* fwd 5’-3’: CAACGGCTCCGGCATGTGC and rev 5’-3’: CTCTTGCTCTGGGCCTCG; *Vdac1* fwd 5’-3’: CAAGGTCACACTGAACATGG and rev 5’-3’: TCACTTTGGTGGTTTCCGT; *Tomm20* fwd 5’-3’: TGCATCTACTTCGACCGCAAA and rev 5’-3’: GTCCACACCCTTCTCGTAGTC; *mt-Co2* fwd 5’-3’: CCTCCACTCATGAGCAGTCC and rev 5’-3’: AATAACCCTGGTCGGTTTG; *mt-Nd1* fwd 5’-3’: CTAGCAGAAACAAACCGGGC and rev 5’-3’: CCGGCTGCGTATTCTACGTT) in the ABI QuantStudio 3 system (Thermo Fisher Scientific). Actin-beta (*Actb*) was used as a reference housekeeping gene. Data analysis was carried out using the 2$^{\wedge }$-ddCt method [98]. Data were visualized with Prism software (GraphPad) and Mann-Whitney nonparametric test was used to compare $rod^{\varDelta \ Vhl}$ and ctrl mice.

## Supplementary information

**Additional file 1: Figure S1.** Light-induced calcium response in rods during TPLSM. **a**, Expression of the calcium sensor GCaMP6s (green) in cone arrestin (cARR, red) negative photoreceptors after subretinal AAV application. White arrows: cARR-positive but GCaMP6s negative cells. **b**, Preparation of acute retinal slices from flat mounted half retinas and imaging of GCaMP6s by TPLSM. **c**, Representative TPLSM micrographs illustrating the drop of intracellular calcium levels during imaging. Left: Max intensity of whole image series. Middle and right: intensity weighted images at 0 s (middle) and 4 s (right) of imaging. White arrows indicate regions with reduced GCaMP6s signal after 4 s of imaging. Scale bars, 50 μm. **d**, Representative GCaMP6s traces in the outer plexiform layer (OPL), outer nuclear layer (ONL), and photoreceptor segments (PS) of wild type mice during 6 s of TPLSM. **e**, GCaMP6s signal (mean $\pm$  SD) in the OPL of *Gnat1a*^*-/-*^ mice (12 retinal slices from 4 mice) at 2, 4, and 6 s during TPLSM.

**Additional file 2: Figure S2.** Variability and mosaicism of Cre levels in $rod^{\varDelta\ Vhl}$ mice. **a**, Relative normalized abundance of Cre, SAG, and GAPDH in the outer nuclear layer (ONL) of $rod^{\varDelta\ Vhl}$ mice at 2.5 months of age. Shown are means (normalized to 1) $\pm$ SD and individual data points. The samples with the highest (red) and lowest (blue) Cre level are indicated. Statistics: Bartlett's test for homoscedasticity. **b**, Immunofluorescence labeling for Cre in $rod^{\varDelta\ Vhl}$ mice at 2.5 months of age. White box: magnification of the ONL region. Scale bar, 50 μm. Panorama: Scale bar, 500 μm.

**Additional file 3: Figure S3.** Expression of mitochondrial genes in the ONL and PS of $rod^{\varDelta\ Vhl}$ mice. **a**, Relative expression of *Vdac1* and *Tomm20* in ONL and PS samples from 2.5 months old $rod^{\varDelta\ Vhl}$ and ctrl mice. **b**, Relative expression of the mt-DNA encoded genes *mt-Co2* and *mt-Nd1* in ONL and PS samples from 2.5 months old $rod^{\varDelta\ Vhl}$ and ctrl mice. *N* = 6 mice per genotype. Statistics: Mann-Whitney nonparametric test.

**Additional file 4: Figure S4.** Retinal function of $rod^{\varDelta\ Cox10}$ mice. **a**, Scotopic single flash ERG responses to light stimuli with increasing intensities (top to bottom) of $rod^{\varDelta\ Cox10}$ (orange) and ctrl (black) mice at 6 (left) and 12 (right) months of age. Shown are averaged traces. **b**,**c**, Scotopic a-wave (**b**) and b-wave (**c**) amplitudes as a function of stimulus intensity derived from (**a**). Shown are means $\pm$ SD. **: p $\leq$ 0.01. ****: p $\leq$ 0.0001. **d**, Photopic single flash ERG responses to light stimuli with increasing intensities (top to bottom) of $rod^{\varDelta\ Cox10}$ (orange) and ctrl (black) mice at 6 (left) and 12 (right) months of age. Shown are averaged traces. **e**, Photopic b-wave amplitudes as a function of stimulus intensity derived from (**d**). Shown are means $\pm$ SD. *N* = 5 (6-month-old $rod^{\varDelta\ Cox10}$ mice); *N* = 6 (6- and 12-month-old ctrl mice; 12-month-old $rod^{\varDelta\ Cox10}$ mice).

**Additional file 5: Table S1.** Top 25 upregulated proteins in the PS and ONL.

**Additional file 6: Table S2.** Top 50 differentially regulated proteins in the ONL of $rod^{\varDelta\ Vhl}$ mice.

**Additional file 7: Table S3.** Top 50 differentially regulated proteins in the PS of $rod^{\varDelta\ Vhl}$ mice.

**Additional file 8: Table S4.** Differentially regulated mitochondrial proteins in the PS of $rod^{\varDelta\ Vhl}$ mice.

**Additional file 9: Table S5.** Proteomic analysis of ONL and PS from $rod^{\varDelta\ Vhl}$ and ctrl mice; includes UniProt IDs, number of unique peptides, ensembl IDs, gene symbols and short description, abundance ratios, adj-p values and raw data.

## Data Availability

The mass spectrometry proteomic data have been deposited to the ProteomeXchange Consortium via the PRIDE partner repository with the dataset identifier PXD034057.

## Abbreviations

AAV: Adeno-associated viruses
ACSF: Artificial cerebrospinal fluid
AMD: Age-related macular degeneration
COX: Cytochrome c oxidase
ERG: Electroretinography
GCL: Ganglion cell layer
GSEA: Gene set enrichment analysis
HIF: Hypoxia-inducible factor
HIFA: HIF-alpha
INL: Inner nuclear layer
IPL: Inner plexiform layer
IS: Photoreceptor inner segments
MCT: Monocarboxylate transporters
mOP: Mouse opsin promoter
MT-CO2: Cytochrome c oxidase subunit 2
mt-Nd1: NADH-ubiquinone oxidoreductase chain 1
ONL: Outer nuclear layer
OPL: Outer plexiform layer
OS: Photoreceptor outer segments
OXPHOS: Oxidative phosphorylation pathway
PCA: Principal component analysis
PR: Photoreceptor
PS: Photoreceptor segments
RPE: Retinal pigmented epithelium
SDH: Succinate dehydrogenase
TCA: Tricarboxylic acid
TFAM: Mitochondrial transcription factor A
Tomm20: Translocase of outer mitochondrial membrane 20
TPLSM: Two-photon laser scanning microscopy
VDAC1: Voltage dependent anion channel 1
VEGF: Vascular endothelial growth factor
VHL: Von Hippel-Lindau tumor suppressor protein
