# Supplementary material for: Deficits in mitochondrial TCA cycle and OXPHOS precede rod photoreceptor degeneration during chronic HIF activation
Source: Mol Neurodegener. 2023 Mar 7;18:15. doi: 10.1186/s13024-023-00602-x (PMC9990367; doi:10.1186/s13024-023-00602-x)
Supplement: Supplementary file 1 — Additional file 1: Figure S1. Light-induced calcium response in rods during TPLSM. a, Expression of the calcium sensor GCaMP6s (green) in cone arrestin (cARR, red) negative photoreceptors after subretinal AAV application. White arrows: cARR-positive but GCaMP6s negative cells. b, Preparation of acute retinal slices from flat mounted half retinas and imaging of GCaMP6s by TPLSM. c, Representative TPLSM micrographs illustrating the drop of intracellular calcium levels during imaging. Left: Max intensity of whole image series. Middle and right: intensity weighted images at 0 s (middle) and 4 s (right) of imaging. White arrows indicate regions with reduced GCaMP6s signal after 4 s of imaging. Scale bars, 50 μm. d, Representative GCaMP6s traces in the outer plexiform layer (OPL), outer nuclear layer (ONL), and photoreceptor segments (PS) of wild type mice during 6 s of TPLSM. e, GCaMP6s signal (mean $\pm$± SD) in the OPL of Gnat1a-/- mice (12 retinal slices from 4 mice) at 2, 4, and 6 s during TPLSM. [file 13024_2023_602_MOESM1_ESM.pdf]

Fig. S1

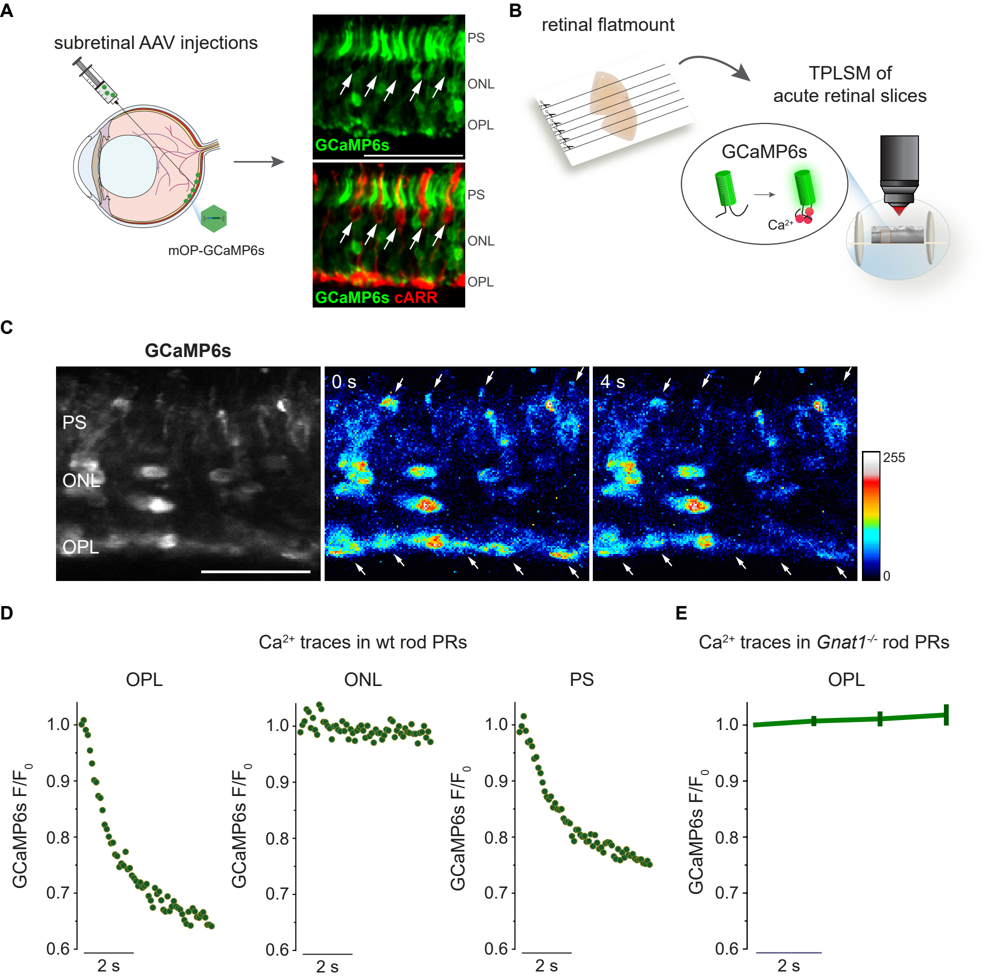

A
subretinal AAV injections
mOP-GCaMP6s
PS
ONL
OPL
GCaMP6s
GCaMP6s cARR
B
retinal flatmount
TPLSM of
acute retinal slices
GCaMP6s
Ca2+
C
GCaMP6s
PS
ONL
OPL
0 s
4 s
255
0
D
Ca2+ traces in wt rod PRs
OPL
ONL
PS
GCaMP6s F/F0
1.0
0.9
0.8
0.7
0.6
2 s
E
Ca2+ traces in Gnat1-/- rod PRs
OPL
GCaMP6s F/F0
1.0
0.9
0.8
0.7
0.6
2 s
