# Supplementary figures and images for: Deficits in mitochondrial TCA cycle and OXPHOS precede rod photoreceptor degeneration during chronic HIF activation
Source: Mol Neurodegener. 2023 Mar 7;18:15. doi: 10.1186/s13024-023-00602-x (PMC9990367; doi:10.1186/s13024-023-00602-x)

**Fig. S2**

**A**

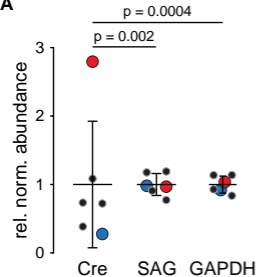

**B**

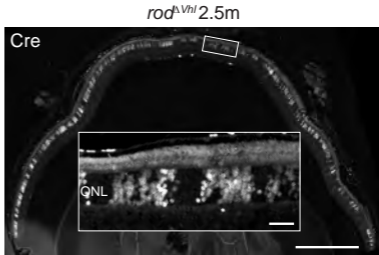

Supplement: Supplementary file 2 — Additional file 2: Figure S2. Variability and mosaicism of Cre levels in $rod^{\varDelta\ Vhl}$rodΔVhl mice. a, Relative normalized abundance of Cre, SAG, and GAPDH in the outer nuclear layer (ONL) of $rod^{\varDelta\ Vhl}$rodΔVhl mice at 2.5 months of age. Shown are means (normalized to 1) $\pm$± SD and individual data points. The samples with the highest (red) and lowest (blue) Cre level are indicated. Statistics: Bartlett's test for homoscedasticity. b, Immunofluorescence labeling for Cre in $rod^{\varDelta\ Vhl}$rodΔVhl mice at 2.5 months of age. White box: magnification of the ONL region. Scale bar, 50 μm. Panorama: Scale bar, 500 μm. [file 13024_2023_602_MOESM2_ESM.pdf]

**Fig. S3****A**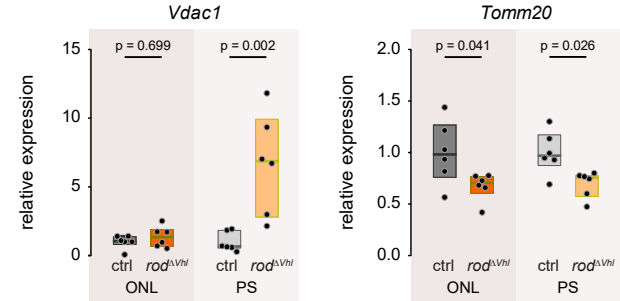**B**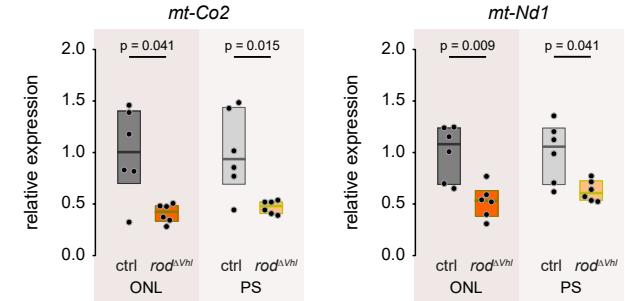

Supplement: Supplementary file 3 — Additional file 3: Figure S3. Expression of mitochondrial genes in the ONL and PS of $rod^{\varDelta\ Vhl}$rodΔVhl mice. a, Relative expression of Vdac1 and Tomm20 in ONL and PS samples from 2.5 months old $rod^{\varDelta\ Vhl}$rodΔVhl and ctrl mice. b, Relative expression of the mt-DNA encoded genes mt-Co2 and mt-Nd1 in ONL and PS samples from 2.5 months old $rod^{\varDelta\ Vhl}$rodΔVhl and ctrl mice. N = 6 mice per genotype. Statistics: Mann-Whitney nonparametric test. [file 13024_2023_602_MOESM3_ESM.pdf]

A

## Scotopic ERG

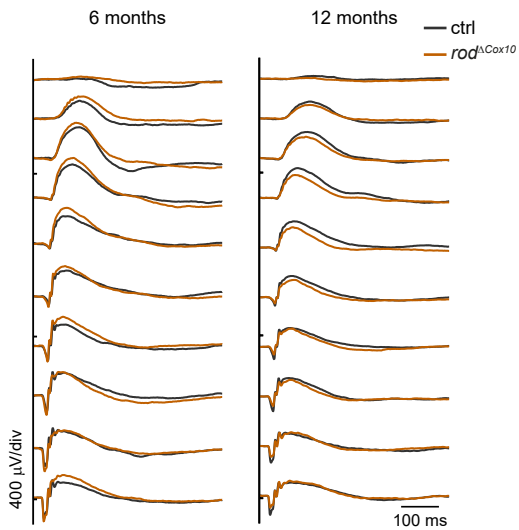

B

## Scotopic a-wave

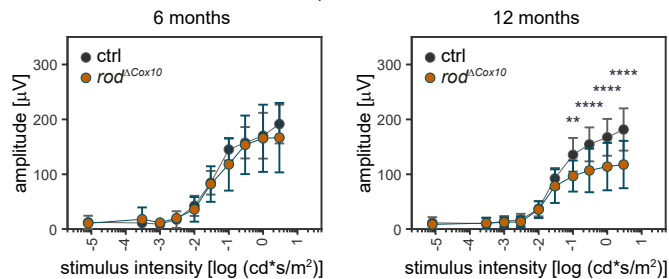

C

## Scotopic b-wave

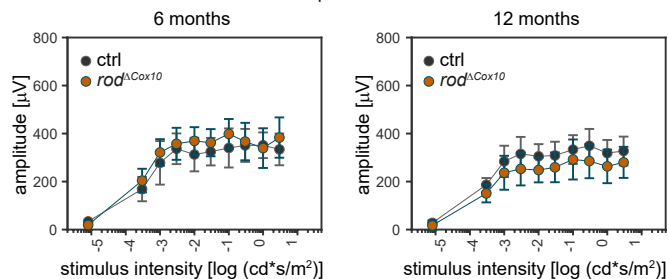

D

## Photopic ERG

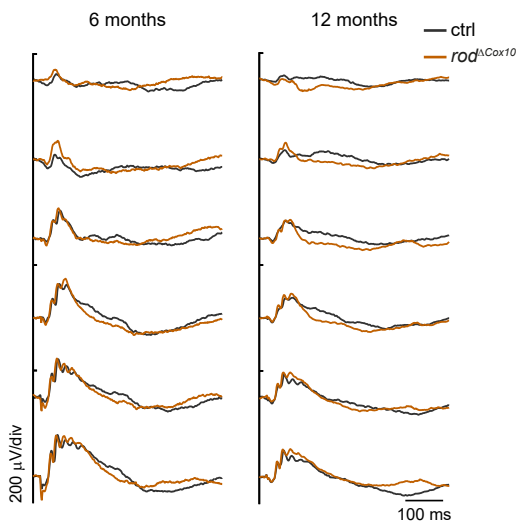

E

## Photopic b-wave

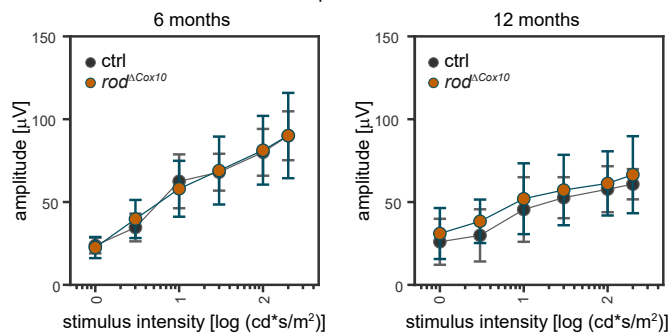

Supplement: Supplementary file 4 — Additional file 4: Figure S4. Retinal function of $rod^{\varDelta\ Cox10}$rodΔCox10 mice. a, Scotopic single flash ERG responses to light stimuli with increasing intensities (top to bottom) of $rod^{\varDelta\ Cox10}$rodΔCox10 (orange) and ctrl (black) mice at 6 (left) and 12 (right) months of age. Shown are averaged traces. b,c, Scotopic a-wave (b) and b-wave (c) amplitudes as a function of stimulus intensity derived from (a). Shown are means $\pm$± SD. **: p $\leq$≤ 0.01. ****: p $\leq$≤ 0.0001. d, Photopic single flash ERG responses to light stimuli with increasing intensities (top to bottom) of [file 13024_2023_602_MOESM4_ESM.pdf]
