# Supplementary material for: Deficits in mitochondrial TCA cycle and OXPHOS precede rod photoreceptor degeneration during chronic HIF activation
Source: Mol Neurodegener. 2023 Mar 7;18:15. doi: 10.1186/s13024-023-00602-x (PMC9990367; doi:10.1186/s13024-023-00602-x)
Supplement: Supplementary file 5 — Additional file 5: Table S1. Top 25 upregulated proteins in the PS and ONL. [file 13024_2023_602_MOESM5_ESM.pdf]

Table S1: Top 25 upregulated proteins in the PS and ONL

Uniprot ID	Gene symbol	Abundance Ratio [PS/ONL]	Adjusted P Value	PS		ONL	
				mean abundance	SD	mean abundance	SD
P32958	Rom1	81.14	0.000001	6.56e+6	2.08e+6	5.32e+8	9.03e+7
P29974	Cnga1 *	42.38	<0.000001	4.15e+6	1.66e+6	1.76e+8	2.28e+7
Q9DC23	Dnajc10	39.74	<0.000001	2.97e+5	2.30e+5	1.18e+7	1.43e+6
P15409	Rho *	34.68	<0.000001	6.19e+7	2.40e+7	2.15e+9	3.36e+8
Q8C1Z7	Bbs4 **	29.93	0.000003	8.57e+5	6.25e+5	2.57e+7	4.81e+6
P51491	Opn1sw *	26.36	0.000034	4.48e+6	1.37e+6	1.18e+8	2.99e+7
O54990	Prom1	26.26	<0.000001	2.43e+6	8.78e+5	6.37e+7	6.46e+6
P23440	Pde6b *	26.12	<0.000001	2.97e+7	1.79e+7	7.77e+8	8.65e+7
P52785	Gucy2e *	23.71	<0.000001	1.66e+7	3.17e+6	3.95e+8	5.35e+7
Q99LE6	Abcf2	22.42	0.000004	1.21e+6	2.78e+5	2.71e+7	5.29e+6
O08600	Endog ***	21.92	<0.000001	2.56e+5	1.21e+5	5.61e+6	6.78e+5
Q791V5	Mtch2	21.42	<0.000001	8.89e+5	4.81e+5	1.90e+7	1.84e+6
Q66GT5	Ptpmt1	21.18	0.003045	2.55e+5	1.61e+5	5.40e+6	2.64e+6
Q8R1W8	Impg1	20.89	<0.000001	2.82e+7	7.05e+6	5.89e+8	7.09e+7
Q148R9	Rgs9bp *	20.35	<0.000001	6.72e+6	8.56e+5	1.37e+8	1.32e+7
O35599	Opn1mw *	18.54	0.00001	3.38e+6	4.68e+5	6.26e+7	1.35e+7
Q8VC33	Nxnl1	17.90	0.000058	3.23e+5	1.71e+5	5.78e+6	1.53e+6
Q9DCZ4	Apoo	14.51	<0.000001	1.23e+6	3.60e+5	1.78e+7	2.62e+6
Q62425	Ndufa4 ***	13.62	0.000147	4.60e+6	2.30e+6	6.27e+7	1.83e+7
Q9CRB9	Chchd3	12.90	0.000182	1.98e+6	6.64e+5	2.55e+7	7.73e+6
P50149	Gnat2 ***	12.20	<0.000001	5.48e+6	1.28e+6	6.68e+7	9.99e+6
Q9CQ75	Ndufa2 ***	11.70	0.002252	4.45e+5	3.73e+5	5.20e+6	2.25e+6
Q8JZN7	Rhot2 ***	11.44	<0.000001	9.61e+6	2.48e+6	1.10e+8	1.35e+7
Q9CPQ8	Atp5mg ***	11.30	0.000237	1.80e+6	8.81e+5	2.03e+7	6.34e+6
P27664	Pde6a *	11.22	<0.000001	1.01e+8	1.87e+7	1.13e+9	1.35e+8
Q91VM5	Rbmxl1 ****	0.03	0.000002	2.14e+7	3.83e+6	5.56e+5	2.92e+5
Q9JLB0	Mpp6	0.05	<0.000001	2.66e+7	3.43e+6	1.42e+6	1.02e+6
Q80X41	Vrk1	0.06	0.000001	1.83e+7	2.86e+6	1.08e+6	7.90e+5
Q6PDQ2	Chd4 ****	0.06	<0.000001	9.46e+7	4.56e+6	5.58e+6	3.48e+6
Q91VL8	Terf2ip ****	0.06	<0.000001	1.37e+7	1.17e+6	8.44e+5	6.56e+5
Q569Z6	Thrap3 ****	0.06	<0.000001	6.80e+7	4.74e+6	4.22e+6	2.95e+6
P10922	H1-0 ****	0.06	<0.000001	7.86e+8	1.05e+8	4.97e+7	3.05e+7
Q6DFW4	Nop58 ****	0.07	<0.000001	5.19e+7	4.07e+6	3.49e+6	1.93e+6
P12658	Calb1	0.07	0.007289	1.27e+7	7.17e+6	8.57e+5	6.59e+5
P58269	Dpf3 ****	0.07	0.000002	1.37e+7	2.26e+6	9.34e+5	7.30e+5
P43247	Msh2 ****	0.07	<0.000001	1.09e+7	8.68e+5	7.89e+5	5.12e+5
Q00422	Gabpa ****	0.07	<0.000001	1.12e+7	9.18e+5	8.10e+5	3.53e+5
Q8VE37	Rcc1	0.07	<0.000001	2.67e+7	2.93e+6	1.96e+6	1.34e+6
Q8VDP4	Ccar2	0.07	<0.000001	4.08e+7	1.66e+6	3.01e+6	2.01e+6
P18608	Hmgn1 ****	0.07	0.000001	6.54e+7	1.03e+7	4.86e+6	2.86e+6
Q3UL36	Arglu1	0.07	0.000003	1.07e+7	1.91e+6	8.02e+5	3.60e+5
Q9JIX8	Acin1 ****	0.07	<0.000001	7.74e+7	5.98e+6	5.80e+6	3.00e+6
P83917	Cbx1	0.08	<0.000001	5.18e+7	3.99e+6	3.89e+6	1.99e+6
Q923G2	Polr2h ****	0.08	<0.000001	5.83e+6	4.67e+5	4.47e+5	2.05e+5
P35550	Fbl ****	0.08	<0.000001	5.09e+7	4.93e+6	3.90e+6	2.50e+6
Q91WJ8	Fubp1 ****	0.08	<0.000001	1.67e+7	1.93e+6	1.29e+6	9.28e+5
Q00899	Yy1 ****	0.08	<0.000001	1.26e+7	1.53e+6	9.77e+5	4.66e+5
P02088	Hbb-b1	0.08	0.002196	3.90e+7	1.64e+7	3.04e+6	3.10e+6
P43276	H1-5 ****	0.08	<0.000001	9.93e+8	1.24e+8	7.75e+7	5.31e+7
Q8BG81	Poldip3 ****	0.08	0.000001	1.28e+7	1.95e+6	1.03e+6	9.11e+5

*proteins belonging to the phototransduction cascade

**proteins belonging to the cilium

***mitochondrial associated proteins

****nuclear proteins
