# Supplementary material for: Deficits in mitochondrial TCA cycle and OXPHOS precede rod photoreceptor degeneration during chronic HIF activation
Source: Mol Neurodegener. 2023 Mar 7;18:15. doi: 10.1186/s13024-023-00602-x (PMC9990367; doi:10.1186/s13024-023-00602-x)
Supplement: Supplementary file 6 — Additional file 6: Table S2. Top 50 differentially regulated proteins in the ONL of $rod^{\varDelta\ Vhl}$rodΔVhl mice. [file 13024_2023_602_MOESM6_ESM.pdf]

**Table S2**: Top 50 differentially regulated proteins in the ONL of *rod*$^{\Delta Vhl}$ mice

| Uniprot ID | Gene symbol | Abundance Ratio [*rod*$^{\Delta Vhl}$/ctrl] | P Value |
|---|---|---|---|
| Q91WT4 | Dnajc17 | 100 | <0.000001 |
| P01901 | H2-K1 | 100 | <0.000001 |
| Q8K4Q0 | Rptor | 100 | <0.000001 |
| Q7TMY4 | Thoc7 | 100 | <0.000001 |
| P01902 | H2-K1 | 20.11 | <0.000001 |
| D3YZP9 | Ccdc6 | 16.52 | <0.000001 |
| Q9CZ28 | Snf8 | 16.36 | <0.000001 |
| Q9Z1P6 | Ndufa7 | 15.39 | <0.000001 |
| P06956 | Cre | 8.32 | <0.000001 |
| P84228 | H3c2 | 7.54 | <0.000001 |
| Q8VEJ9 | Vps4a | 5.30 | <0.000001 |
| P32037 | Slc2a3* | 5.10 | <0.000001 |
| Q9CW07 | Ppp1r3g | 4.38 | <0.000001 |
| P60762 | Morf4l1 | 3.05 | 0.0001 |
| Q8R1F0 | D8Ertd738e | 2.99 | <0.000001 |
| Q3TGF2 | Fam107b | 2.98 | 0.000004 |
| Q9CQS2 | Nop10 | 2.92 | 0.0031 |
| P56812 | Pdcd5 | 2.89 | 0.000001 |
| Q8K442 | Abca8a | 2.83 | <0.000001 |
| Q64522 | H2ac21 | 2.61 | <0.000001 |
| P51880 | Fabp7 | 2.55 | 0.00003 |
| Q8VHR5 | Gatad2b | 2.53 | 0.00004 |
| Q9CY57 | Chtop | 2.47 | <0.000001 |
| Q8C6B9 | Rps19bp1 | 2.41 | 0.0022 |
| Q9CQB5 | Cisd2 | 2.37 | 0.00005 |
| Q9DAI2 | Ift22 | 0.36 | 0.000005 |
| Q99M74 | Krt82 | 0.33 | <0.000001 |
| Q9QYB1 | Clic4 | 0.29 | <0.000001 |
| O70494 | Sp3 | 0.28 | <0.000001 |
| Q8VED5 | Krt79 | 0.27 | <0.000001 |
| Q61414 | Krt15 | 0.27 | <0.000001 |
| Q7TND5 | Rpf1 | 0.10 | <0.000001 |
| Q5F293 | Zbtb4 | 0.10 | <0.000001 |
| Q8BSZ2 | Ap3s2 | 0.10 | <0.000001 |
| Q9JM96 | Cdc42ep4 | 0.09 | <0.000001 |
| Q9JI90 | Rnf14 | 0.09 | <0.000001 |
| Q6PAV2 | Herc4 | 0.09 | <0.000001 |
| O70172 | Pip4k2a | 0.08 | <0.000001 |
| Q8CFC2 | Myt1 | 0.07 | <0.000001 |
| Q8BWW9 | Pkn2 | 0.07 | <0.000001 |
| O70480 | Vamp4 | 0.06 | <0.000001 |
| Q3UDR8 | Yipf3 | 0.06 | <0.000001 |
| Q8R3V5 | Sh3glb2 | 0.06 | <0.000001 |
| Q8BH24 | Tm9sf4 | 0.05 | <0.000001 |
| Q03137 | Epha4 | 0.01 | <0.000001 |
| Q91WK5 | Gcsh | 0.01 | <0.000001 |
| Q6Y7W8 | Gigyf2 | 0.01 | <0.000001 |
| Q8R3H7 | Hs2st1 | 0.01 | <0.000001 |
| Q99L88 | Sntb1 | 0.01 | <0.000001 |
| Q811S7 | Ubp1 | 0.01 | <0.000001 |

*glycolysis associated proteins
