# Supplementary material for: Deficits in mitochondrial TCA cycle and OXPHOS precede rod photoreceptor degeneration during chronic HIF activation
Source: Mol Neurodegener. 2023 Mar 7;18:15. doi: 10.1186/s13024-023-00602-x (PMC9990367; doi:10.1186/s13024-023-00602-x)
Supplement: Supplementary file 7 — Additional file 7: Table S3. Top 50 differentially regulated proteins in the PS of $rod^{\varDelta\ Vhl}$rodΔVhl mice. [file 13024_2023_602_MOESM7_ESM.pdf]

Table S3: Top 50 differentially regulated proteins in the PS of $rod^{\Delta Vhl}$ mice

Uniprot ID	Gene symbol	Abundance Ratio [$rod^{\Delta Vhl}$/ctrl]	P Value
Q8BK35	Nop53	100	<0.000001
P07356	Anxa2	20.26	<0.000001
P32037	Slc2a3*	9.77	<0.000001
Q3V3R1	Mthfd1l	4.04	<0.000001
Q9CW07	Ppp1r3g	3.56	<0.000001
Q6RT24	Cenpe	3.09	<0.000001
O35143	ATP5IF1	3.04	<0.000001
P57787	Slc16a3*	2.88	<0.000001
P51880	Fabp7	2.39	0.000013
P17156	Hspa2	2.37	<0.000001
O54879	Hmgb3	2.26	<0.000001
Q91YE3	Egln1	2.24	0.000001
P84228	H3c2	2.20	0.000198
Q9R0N0	Galk1	2.18	<0.000001
Q9Z2F7	Bnip3l	2.08	0.0044
P21956	Mfge8	2.04	<0.000001
Q69Z98	Brsk2	2.00	0.0077
P29788	Vtn	1.98	0.000026
Q62313	Tgoln1	1.97	0.001
Q61414	Krt15	1.96	0.000002
Q9R099	Tbl2	1.94	<0.000001
Q06890	Clu	1.92	0.000001
Q91ZR1	Rab4b	1.90	0.047
Q922U2	Krt5	1.90	0.000001
Q9CQZ1	Hsbp1	1.89	0.00033
Q9JJE4	Paqr4	0.52	0.004
Q8VBV8	Guca1b	0.52	<0.000001
P85094	Isoc2a	0.50	0.000003
Q9JM76	Arpc3	0.50	0.000069
Q9DCX2	Atp5pd	0.49	<0.000001
P39054	Dnm2	0.49	0.000007
B9EJ86	Osbpl8	0.47	0.0024
Q9JJU9	Crybb3	0.46	0.00046
P62696	Crybb2	0.44	<0.000001
P24622	Cryaa	0.44	<0.000001
Q62048	Pea15	0.42	<0.000001
Q9WVL0	Gstz1	0.42	<0.000001
Q91ZQ1	Pde6c	0.42	<0.000001
P99028	Uqcrh	0.39	0.000053
Q9JJV1	Cryba2	0.39	0.000038
Q91W97	Hkdc1*	0.39	0.000004
Q9DCU6	Mrpl4	0.36	0.00008
P04344	Crygb	0.18	<0.000001
Q8K2I1	Fntb	0.10	<0.000001
P61028	Rab8b	0.10	<0.000001
Q8BV13	Cops7b	0.01	<0.000001
P02525	Cryba1	0.01	<0.000001
O35943	Fxn	0.01	<0.000001
P58044	Idi1	0.01	<0.000001
Q9D6S7	Mrrf	0.01	<0.000001

*glycolysis associated proteins
