# Supplementary material for: Deficits in mitochondrial TCA cycle and OXPHOS precede rod photoreceptor degeneration during chronic HIF activation
Source: Mol Neurodegener. 2023 Mar 7;18:15. doi: 10.1186/s13024-023-00602-x (PMC9990367; doi:10.1186/s13024-023-00602-x)
Supplement: Supplementary file 8 — Additional file 8: Table S4. Differentially regulated mitochondrial proteins in the PS of $rod^{\varDelta\ Vhl}$rodΔVhl mice. [file 13024_2023_602_MOESM8_ESM.pdf]

Table S4: Differentially regulated mitochondrial proteins in the PS of $rod^{\Delta Vhl}$ mice

Uniprot ID	Gene symbol	Abundance Ratio [$rod^{\Delta Vhl}$/ctrl]	P Value
O55003	Bnip3	100	<0.000001
Q3V3R1	Mthfd1l	4.04	<0.000001
O35143	ATP5IF1	3.04	<0.000001
Q9Z2F7	Bnip3l	2.08	0.004
Q922H2	Pdk3	1.48	0.013
P09528	Fth1	1.41	0.014
Q8R4N0	Clybl	1.39	0.047
Q9D6U8	Fam162a	1.37	0.024
Q91Z53	Grhpr	1.31	0.034
Q9DC29	Abcb6	0.80	0.042
Q925I1	Atad3	0.78	0.029
Q99JB2	Stoml2	0.77	0.040
Q9CQZ6	Ndufb3	0.77	0.022
Q91VN4	Chchd6	0.76	0.024
P99029	Prdx5	0.76	0.043
Q8CIM3	D2hgdh	0.74	0.039
P52503	Ndufs6	0.74	0.012
Q921H8	Acaa1a	0.74	0.006
Q9D0M3	Cyc1	0.73	0.024
Q8BK08	Tmem11	0.73	0.024
Q9CR21	Ndufab1	0.73	0.029
Q9CZU6	Cs	0.73	0.022
Q9CQJ8	Ndufb9	0.73	0.007
P47740	Aldh3a2	0.72	0.006
Q9CRB9	Chchd3	0.72	0.010
Q9DCS3	Mecr	0.71	0.004
Q9DCS9	Ndufb10	0.71	0.005
Q99L13	Hibadh	0.71	0.011
P00405	Mtco2	0.70	0.009
Q9CYK1	Wars2	0.70	0.042
Q8C163	Exog	0.69	0.012
Q9WTP6	Ak2	0.69	0.003
Q9JLZ3	Auh	0.68	0.006
Q9WTP7	Ak3	0.68	0.003
Q9CQ69	Uqcrq	0.68	0.002
Q9CPQ8	Atp5mg	0.68	0.002
Q66GT5	Ptpmt1	0.68	0.006
P12787	Cox5a	0.68	0.004
Q8BWM0	Ptges2	0.67	0.001
P97493	Txn2	0.66	0.004
P19783	Cox4i1	0.66	0.002
Q91ZE0	Tmlhe	0.65	0.0005
Q3UUI3	Them4	0.65	0.002
Q99LP6	Grpel1	0.65	0.002
P56380	Nudt2	0.65	0.016
Q924T2	Mrps2	0.64	0.026
Q9CPQ1	Cox6c	0.63	0.0001
Q9CQ75	Ndufa2	0.63	0.001
Q9QZM2	Polg2	0.62	0.007
P62897	Cycs	0.62	0.0005
Q9CQQ7	Atp5pb	0.62	0.0004
Q9CQ54	Ndufc2	0.61	0.001
Q9DCT2	Ndufs3	0.60	0.0002
Q9CPP6	Ndufa5	0.60	0.0004
Q9CQC7	Ndufb4	0.59	0.001
Q8BHC4	Dcakd	0.59	0.001
Q9CQZ5	Ndufa6	0.58	0.000004
P56135	Atp5mf	0.58	0.00004
Q9DB20	Atp5po	0.57	0.00003
P20108	Prdx3	0.57	0.00003
Q9DCJ5	Ndufa8	0.56	0.000002
Q8R2Y8	Ptrh2	0.55	0.0001
Q9D1H6	Ndufaf4	0.55	0.004
Q9Z1P6	Ndufa7	0.54	0.00005
Q8R1I1	Uqcr10	0.54	0.00003
P85094	Isoc2a	0.50	0.000003
Q9DCX2	Atp5pd	0.49	<0.000001
Q9WVL0	Gstz1	0.42	<0.000001
P99028	Uqcrh	0.39	0.00005
Q9DCU6	Mrpl4	0.36	0.00008
O35943	Fxn	0.01	<0.000001
P58044	Idi1	0.01	<0.000001
Q9D6S7	Mrrf	0.01	<0.000001
